# Integrating services for HIV and multidrug-resistant tuberculosis: A global cross-sectional survey among ART clinics in low- and middle-income countries

**DOI:** 10.1371/journal.pgph.0000180

**Published:** 2022-03-01

**Authors:** Kathrin Zürcher, Samyra R. Cox, Marie Ballif, Leslie A. Enane, Olivier Marcy, Marcel Yotebieng, Gary Reubenson, Worarat Imsanguan, Larissa Otero, Nishi Suryavanshi, Stephany N. Duda, Matthias Egger, Jeffrey A. Tornheim, Lukas Fenner, International Epidemiology Databases to Evaluate AIDS (IeDEA)

**Affiliations:** 1 Institute of Social and Preventive Medicine, University of Bern, Bern, Switzerland,; 2 Division of Infectious Diseases, Center for Clinical Global Health Education, Johns Hopkins University School of Medicine, Baltimore, MD, United States of America,; 3 The Ryan White Center for Pediatric Infectious Disease and Global Health, Department of Pediatrics, Indiana University School of Medicine, Indianapolis, IN, United States of America,; 4 U1219 Bordeaux Population Health Research Center, University of Bordeaux, Inserm, IRD, Bordeaux, France,; 5 Division of General Internal Medicine, Department of Medicine, Albert Einstein College of Medicine, Bronx, NY, United States of America,; 6 Faculty of Health Sciences, Department of Paediatrics and Child Health, University of the Witwatersrand, Rahima Moosa Mother and Child Hospital, Johannesburg, South Africa,; 7 Chiangrai Prachanukroh Hospital, Chiang Rai, Thailand,; 8 Instituto de Medicina Tropical Alexander von Humboldt, Universidad Peruana Cayetano Heredia, Lima, Peru,; 9 Facultad de Medicina, Universidad Peruana Cayetano Heredia, Lima, Peru,; 10 Johns Hopkins India, Pune, India,; 11 Department of Biomedical Informatics, Vanderbilt University School of Medicine, Nashville, TN, United States of America,; 12 Centre for Infectious Disease Epidemiology & Research, School of Public Health & Family Medicine, University of Cape Town, Rondebosch, Western Cape, South Africa,; 13 Population Health Sciences, Bristol Medical School, University of Bristol, Bristol, United Kingdom

## Abstract

Tuberculosis (TB) is the leading cause of death among PLHIV and multidrug-resistant-TB (MDR-TB) is associated with high mortality. We examined the management for adult PLHIV coinfected with MDR-TB at ART clinics in lower income countries. Between 2019 and 2020, we conducted a cross-sectional survey at 29 ART clinics in high TB burden countries within the global IeDEA network. We used structured questionnaires to collect clinic-level data on the TB and HIV services and the availability of diagnostic tools and treatment for MDR-TB. Of 29 ART clinics, 25 (86%) were in urban areas and 19 (66%) were tertiary care clinics. Integrated HIV-TB services were reported at 25 (86%) ART clinics for pan-susceptible TB, and 14 (48%) clinics reported full MDR-TB services on-site, i.e. drug susceptibility testing [DST] and MDR-TB treatment. Some form of DST was available on-site at 22 (76%) clinics, while the remainder referred testing off-site. On-site DST for second-line drugs was available at 9 (31%) clinics. MDR-TB treatment was delivered on-site at 15 (52%) clinics, with 10 individualizing treatment based on DST results and five using standardized regimens alone. Bedaquiline was routinely available at 5 (17%) clinics and delamanid at 3 (10%) clinics. Although most ART clinics reported having integrated HIV and TB services, few had fully integrated MDR-TB services. There is a continued need for increased access to diagnostic and treatment options for MDR-TB patients and better integration of MDR-TB services into the HIV care continuum.

## Introduction

Tuberculosis (TB) is the leading cause of death among people living with HIV (PLHIV) [1]. In 2019, PLHIV accounted for about 8% of incident TB patients and 15% of TB mortality globally [1]. Compared to HIV-negative individuals, PLHIV have an increased risk of developing active TB disease, even when on antiretroviral therapy (ART) [2,3]. There is also evidence of an association between HIV and drug-resistant TB; outbreaks involving PLHIV have been well-documented in high HIV burden countries [4,5].

Multidrug-resistant tuberculosis (MDR-TB) challenges global TB control and is associated with high mortality [4]. Strategies for controlling MDR-TB include drug susceptibility testing (DST) to guide treatment and the completion of an adequate treatment regimen. However, managing MDR-TB is difficult, particularly in low- and middle-income countries (LMICs), where there is limited access to both DST to diagnose MDR-TB as well as effective MDR-TB drugs to optimally treat MDR-TB [6,7].

For PLHIV coinfected with MDR-TB, effective coordination among services is particularly important given the complexity of second-line anti-TB treatment and the need for a careful monitoring of side effects. The integration of HIV and TB services has been identified as a global priority, [8,9] especially in regions where both diseases are widespread, which include sub-Saharan Africa, Asia and Latin America. In a multi-regional study conducted in 2012 among 47 ART clinics, we showed that only 26% offered integrated HIV-TB services, with large regional disparities [10]. A study from Uganda showed that integrated HIV and TB services was associated with reduced mortality compared to clinics with no HIV and TB integration [11]. However, it is unclear to what extent integrated services included the management of drug-resistant TB, especially MDR-TB, among PLHIV. Therefore, we conducted a multi-regional survey assessing the capacity and routine practices of ART clinics related to the diagnosis and treatment of MDR-TB among PLHIV.

## Methods

### Study design

This cross-sectional survey was conducted within the International Epidemiology Databases to Evaluate AIDS (IeDEA, www.iedea.org) network, a large consortium of ART clinics pre-dominantly located in LMICs [12,13]. ART clinics within IeDEA are mostly public facilities but are often supported by non-governmental organizations or academic institutions. ART clinics from the Asia-Pacific, Africa, and South America IeDEA regions located in countries defined by the World Health Organization (WHO) as high MDR-TB, high TB, or high TB/HIV burden and countries with TB incidence rates of ≥ 20/100,000 population were eligible to participate were eligible to participate [14]. In this analysis, we focused on the management of adults living with HIV coinfected with MDR-TB.

### Ethics statement

The Cantonal Ethics Committee Bern (Switzerland), Vanderbilt University Medical Center IRB (Tennessee, USA), and Johns Hopkins University School of Medicine IRB (Maryland, USA) approved this project. All participating sites obtained approvals from their local institutional review boards or ethics committees to participate in IeDEA research. All participants provided written informed consent before participating.

### Data collection

IeDEA representatives from each region and an advisory group of TB experts were involved in developing the survey tool. The survey was available in English and French and pilot-tested in both languages by the advisory group of TB experts and selected clinics. Survey data were electronically collected and managed using REDCap (Research Electronic Data Capture) [15,16]. The English version of the online questionnaire is shown in the Supporting Information (S1 Table). All survey respondents were health care workers (HCWs) involved in managing TB patients as clinicians (medical doctors, clinical officers or nurses). The survey consisted of three components: (A) basic information on the ART clinic, including level of care, size, adult/paediatric services, cost of services, and infection control measures; (B) information about the management of adult TB, including case definitions, and availability of diagnostic tools; (C) hypothetical clinical scenarios presenting cases of adult patients co-infected with TB/HIV to assess the clinical practices of treating HCWs. Components A and B were completed by one respondent per clinic. Component C was completed by two to three different respondents per clinic who were asked to respond based on routine practices. The respondent was required to be a HCW at the ART facility who provided care to TB patients (e.g. medical doctor, clinical officer, nurse, etc.) and could consult with others to answer questions. Data collection took place between September 2019 and March 2020.

### Definitions

We defined MDR-TB as resistance to both isoniazid and rifampicin, and extensively drug-resistant TB (XDR-TB) as MDR plus resistance to any fluoroquinolone and at least one second-line injectable drug [17]. We defined an ART clinic with integrated HIV-TB basic services as a clinic where: 1) HIV-positive people are actively screened for TB at enrollment using symptom screening; 2) TB and HIV clinical services are located in the same facility, under the same roof, or available with same-day appointments; and 3) facilities have a specialized clinic/ward on-site with dedicated staff for patients with TB [10]. We categorized the degrees of service integration for MDR-TB as follows: i) *full integration* if both DST and MDR-TB treatment were available on-site, ii) *partial integration*, if either DST or MDR-TB treatment was available on-site, and iii) *off-site services* if both DST and MDR-TB treatment were available off-site. Clinical officers were defined as trained non-physician clinicians and medical doctors as physicians with a university degree.

Any of the following were considered initial TB diagnostic tools: smear microscopy; chest x-ray; any GeneXpert including Xpert MTB/RIF, Ultra, Omni, or XDR (Cepheid, USA); any line probe assay (LPA) including Genotype MTBDRplus or Genotype MTBDRsl (Hain Life-science GmbH, Germany); mycobacterial liquid or solid culture; or urine lipoarabinomannan (LAM). DST was categorised as either molecular (any GeneXpert or any LPA) or phenotypic (mycobacterial culture). The injectable-containing short regimen (also known as the “Bangladesh regimen”) is a standardized short course MDR-TB treatment regimen of 9 to 12 months with or without minor modifications. It consists of an initial 4–6 months of kanamycin, moxifloxacin, ethionamide/prothionamide, clofazimine, pyrazinamide, high-dose isoniazid, and ethambutol, followed by 5 months of moxifloxacin, clofazimine, pyrazinamide, and ethambutol [18,19].

### Analyses

We described ART clinics by the degree of service integration, by region and explored the availability of on-site versus off-site diagnostic tools and treatment for MDR-TB. We analyzed differences among ART clinics using chi-square or Fisher’s exact. Using descriptive statistics, we assessed routine practices related to MDR-TB management captured by hypothetical clinical scenarios. All analyses were done in Stata version 15.1 (Stata Corporation, College Station, TX, USA).

## Results

### Description of the ART clinics

**Fig 1.**
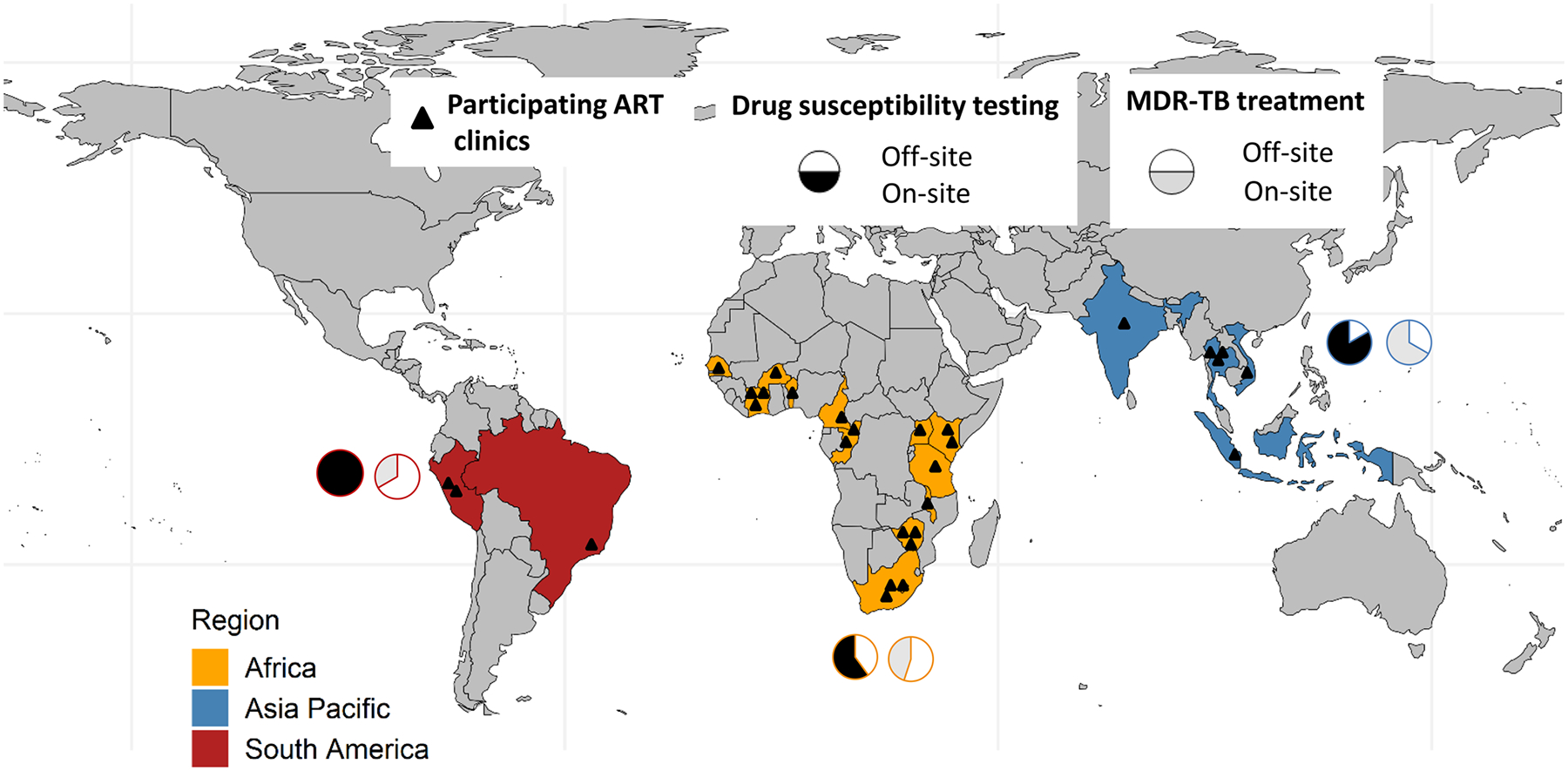
Map showing the participating ART clinics by regions. Pie charts indicate the proportion of clinics with drug-susceptibility testing (DST) (black) and MDR-TB treatment (grey) available on-site by region. Types of DST available included Xpert MTB/RIF, line probe assay, and culture. Abbreviations: MDR, multidrug resistance; TB, Tuberculosis. https://doi.org/10.1371/journal.pgph.0000180.g001

We collected data from 29 adult ART clinics across 19 high TB burden countries (Fig 1). Of 29 ART clinics, 20 (69.0%) were in Africa, 6 (20.7%) were in Asia-Pacific, and 3 (10.3%) were in South America. Clinic-level survey components were completed by medical doctors (23/29, 79.3%), clinical officers (4/29, 13.8%), and nurses (2/29, 6.9%). The majority of respondents (24, 88.9%) had worked at the participating ART clinic for more than five years at the time of survey completion. Overall, 25/29 (86.2%) participating clinics were in urban and 4/29 (13.8%) were in rural settings. Most participating sites (19, 65.5%) were tertiary care clinics (Table 1).

Almost all ART clinics (27, 93.1%) reported following their national TB guidelines to screen, diagnose and treat TB patients. In 2018, the number of people newly diagnosed with MDR-TB at the ART clinics ranged from a few patients to more than 100 patients. For infection control, environmental measures, such as regular natural ventilation, were reported to be in place at 26 (89.8%) of the ART clinics, 19 clinics (65.5%) reported that staff regularly wore masks when in close contact with TB patients or people with presumptive TB (S1 Table), and 18 (62.1%) conducted TB screening among clinic staff who were in contact with TB patients or people with presumptive TB.

### Integration of HIV and MDR-TB services

**Fig 2.**
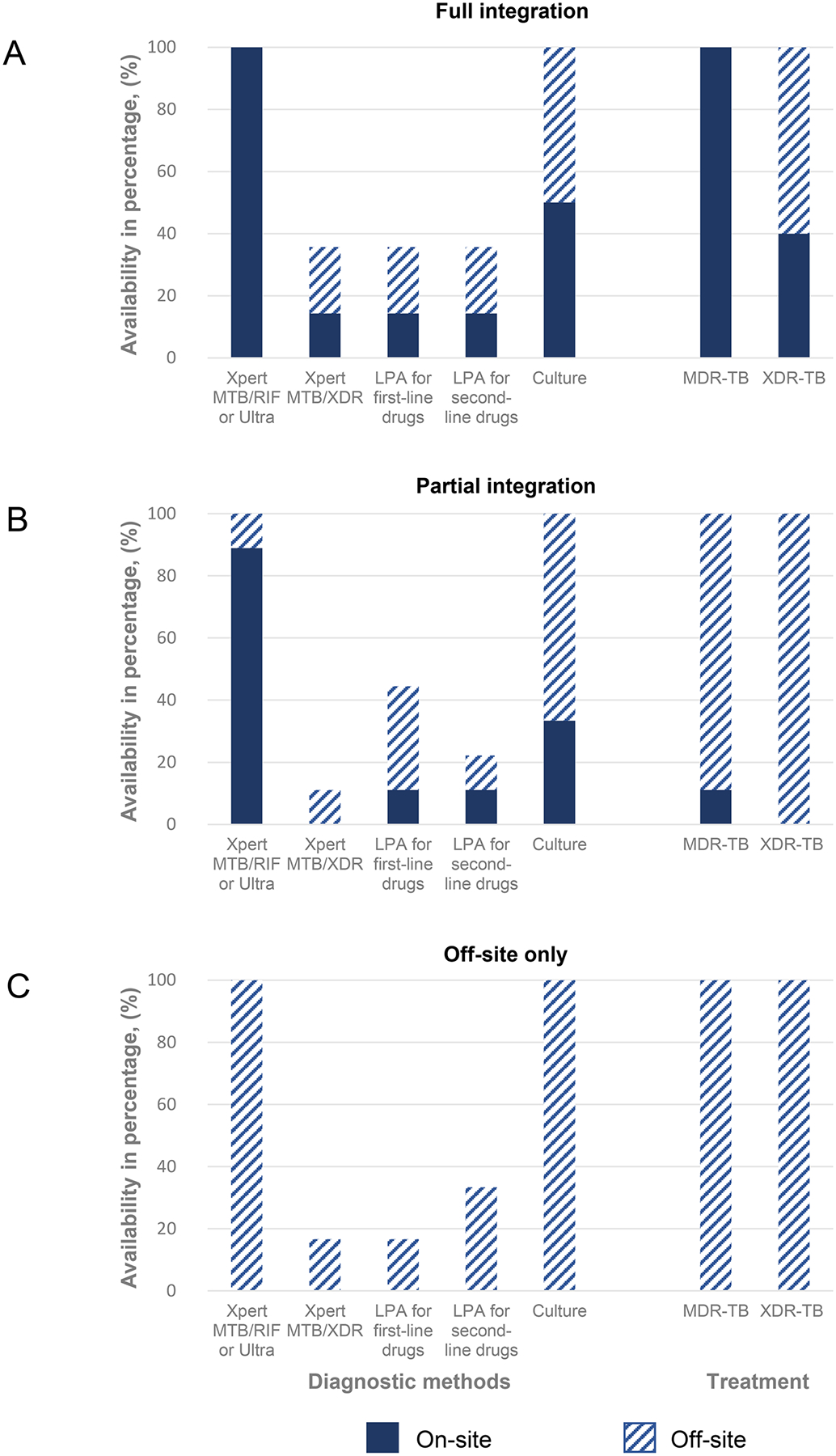
Models of integration of HIV and MDR-TB care. Models of integration of HIV and MDR-TB care. **Panel A**: Fourteen clinics with full integration of DST and MDR-TB treatment on-site; **Panel B**: Partial integration of eight clinics with DST on-site and MDR-TB treatment off-site and one clinic with the opposite; and **Panel C**: Both DST and MDR-TB treatment available off-site only at six clinics. The solid bars represent the percentage of the different molecular or phenotypic DST or MDR/XDR-TB treatment available on-site. The dashed bars represent the percentage of DST or MDR/XDR-TB treatment available off-site. Abbreviations: DST, drug susceptibility testing; MDR, multidrug resistance; TB, Tuberculosis; XDR, extensive drug resistance. https://doi.org/10.1371/journal.pgph.0000180.g002

The majority of participating clinics (25, 86.2%) reported offering integrated HIV-TB basic services, with 25 clinics (86.2%) offering initial TB diagnosis and 20 clinics (69.0%) first-line TB treatment on-site (Table 1). Fourteen clinics (48.3%) reported full integration of HIV and MDR-TB services and nine (31.0%) reported partial integration, of which eight offered on-site DST only and one offered on-site MDR-TB treatment only. Six clinics (20.7%) had access to off-site MDR-TB services only for both diagnosis and treatment (Fig 2, S3 Table).

**Fig 3.**
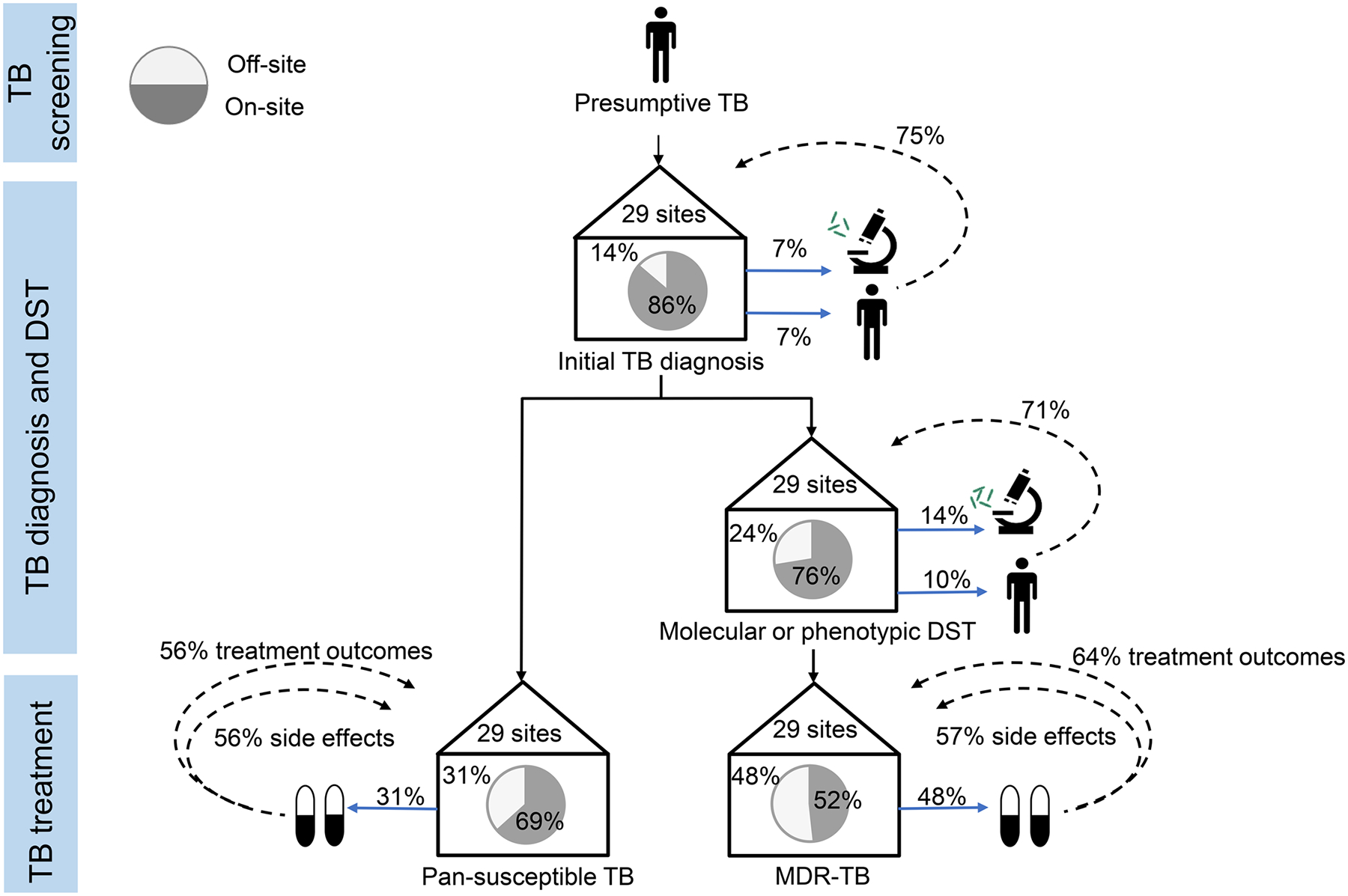
Care pathway for patients with HIV-TB coinfection at 29 ART clinics. Solid blue lines indicate patients or samples sent off-site for TB diagnosis, drug susceptibility testing (DST), or treatment. Dotted lines indicate feedback loops when diagnostic results, DST results, side effect or treatments outcomes are directly communicated back to the ART clinic by phone, written correspondence, or shared database entry. Abbreviations: DST, drug susceptibility testing; MDR, multidrug resistance; TB, Tuberculosis; XDR, extensive drug resistance. https://doi.org/10.1371/journal.pgph.0000180.g003

We describe care pathway for patients coinfected with TB/HIV at participating clinics in Fig 3, from the identification of presumptive TB to drug resistance testing and treatment. Initial TB diagnosis was conducted off-site in four clinics (13.8%), of which half referred patients while the other half sent samples for off-site testing. Three out of four of these clinics received initial TB diagnostic result from the off-site clinic. For any type of DST, seven clinics (24.1%) relied on off-site services, of which three referred patients and four sent samples for off-site testing. Five out of seven of these clinics (71.4%) received DST results from the referral clinic. Treatment of pan-susceptible TB was prescribed off-site in nine clinics (31.0%), of which five (55.6%) received treatment outcome reports from the off-site clinic. For MDR-TB treatment, fourteen clinics (48.3%) referred patients off-site, of which nine (64.3%) received treatment outcome reports for from the referral clinic (Fig 3).

### Drug susceptibility testing

DST, in some form, was available on-site at 22/29 (75.9%) ART clinics. Ten ART clinics reported having molecular and phenotypic DST available on-site, whereas the remaining 12 ART clinics reported having molecular DST only (S4 Table). Molecular DST was most frequently available on-site in South America (3/3, 100%) followed by Asia-Pacific (5/6, 83.3%) and Africa (14/20, 70.0%). In contrast, phenotypic DST was most frequently available on-site in Asia-Pacific (5/6, 83.3%), followed by South America (2/3, 66.7%) and Africa (3/20, 15.0%) (Table 1). Two ART clinics (6.9%) reported access to sequencing technologies.

Mycobacterial culture was available on-site at 10/29 (34.5%) clinics for identifying resistance to first-line drugs. All clinics with some form of DST on-site had a rapid molecular DST for first-line drugs available (22/29, 72.4%). Specifically, Xpert MTB/RIF was available on-site at 21/29 (72.4%), Xpert Ultra at 7/29 (24.1%), and first-line LPA (MTBDRplus) at 3/29 (10.3%, S4 Table). Five (17.2%) ART clinics reported any GeneXpert cartridge stock-outs in the preceding 12 months. In the hypothetical clinical scenario of a patient whose TB treatment is failing, 57/72 (79.2%) HCWs would request a rapid molecular DST to identify resistance to first-line drugs (S5 Table, Scenario 1). The proportion of HCWs who selected this response was higher among ART clinics with full integration of MDR-TB services (31/35, 88.6%) and lower among ART clinics with partial integration and only off-site MDR-TB services (17/24, 70.8%, and 9/13, 69.2%, respectively).

In contrast to first-line DST, DST for second-line drugs was available on-site at 9/29 (31.0%) clinics. Mycobacterial culture for second-line drugs was available at 8/29 (27.6%) clinics; rapid molecular DST for second-line drugs was rarely available. Specifically, Xpert MTB/XDR was available on-site at 2/29 (6.9%) clinics and second-line LPA (MTBDRsl) at 3/29 (10.3%) clinics (S3 and S4 Tables).

### Treatment of MDR-TB

MDR-TB treatment was reported to be offered on-site at 15/29 (51.7%) ART clinics—83.3% of clinics in Asia-Pacific, 45.0% of clinics in Africa, and 33.3% of clinics in South America (Tables 1 and 2). When treated on-site, MDR-TB treatment was most frequently prescribed by medical doctors (10/15, 66.7%), followed by clinical officers (3/15, 20.0%), and nurses (2/15, 13.3%).

The remaining 14 ART clinics (48.3%) referred their MDR-TB patients off-site for MDR-TB treatment. When treated off-site, MDR-TB patients were referred to specific TB clinics (9/14, 64.3%), 6/14 (42.9%) to tertiary care facilities or 3/14 (21.4%) to secondary care facilities. Most referral clinics were located more than 20 km away from the ART clinic (12/14, 85.7%). However, more than half of ART clinics received reports from the referral clinic about their patients’ treatment outcomes (9/14, 64.3%) and side effects (8/14, 57.1, Fig 3).

Overall, 44.8% (13/29) of clinics reported that MDR-TB treatment was individualized, whereas 37.9% (11/29) reported only following standardized and pre-determined MDR-TB treatment protocols, and the remaining reported both individualized and standardized MDR-TB treatment (Table 2). In ART clinics with full integration of MDR-TB services, MDR-TB treatment was most frequently individualized (10/14, 71.4%), whereas in ART clinics with off-site services only, MDR-TB treatment was generally standardized (5/6, 83.3%, S3 Table). Similarly, overall on-site MDR-TB treatment was more frequently individualized than standardized (Table 2). However, all national TB guidelines for participating clinics mentioned a standardized short MDR-TB regimen and most of them a standardized long MDR-TB regimen. Specifically, the standardized injectable-containing short regimen was mentioned in 13/19 (68.4%) national TB guidelines and reported to be in use at 18/29 (62.1%) ART clinics. Additionally, in the hypothetical clinical scenario of a newly diagnosed patient with HIV and rifampicin-resistant TB, 58/72 (80.6%) HCWs would start the patient on a standardised MDR-TB regimen as soon as possible (S5 Table, Scenario 2). All HCWs from ART clinics with full integration selected this response (35/35, 100%), while 17/24 (70.8%) HCWs from partially integrated and 6/13 (46.2%) HCWs from ART clinics with only off-site MDR-TB services selected this option.

DOT was recommended during the entire MDR-TB treatment course at 24/29 (82.8%) ART clinics, whereas 5/29 (17.2%) clinics recommended it only during the intensive phase. Table 2 presents the different types of DOT strategies. XDR-TB treatment was available on-site at 7/29 (24.1%) ART clinics. Among the seven ART clinics that reported treating XDR-TB patients, six used individualized regimens (85.7%). Bedaquiline was routinely available at 5/29 (17.2%) clinics and upon request at another seven (24.1%); delamanid was routinely available at 3/29 (10.3%) clinics and upon request at another seven clinics (24.1%, Table 2).

## Discussions

The integration of TB services into HIV care is key to TB control, especially in regions where the burden of both diseases is high. Yet, little is known about the management of MDR-TB at ART clinics in LMICs. We surveyed 29 ART clinics across three continents about referral practices and the integration of HIV and MDR-TB services. About half of them offered full MDR-TB services on-site, and about three-quarters had at least access to rapid molecular testing for MDR-TB. A fifth of the clinics entirely relied on off-site services to which presumptive MDR-TB patients were referred. We observed substantial regional differences in the management of MDR-TB at ART clinics.

To improve quality of care, ART clinics must strengthen the integration of HIV-TB services [9]. There is evidence that full HIV-TB integration improves HIV and TB care [20]. Studies found a higher proportion of TB treatment success and lower mortality during TB treatment among integrated clinics than non-integrated clinics [11,21]. Inadequate referral mechanisms and poor communication can hamper integrated HIV-TB services in LMICs in settings with partial integration, whereas limited human resources, training, and infrastructure affect settings with full integration [22]. Our findings highlight the challenge of coordinating care when MDR-TB services are partially or entirely performed off-site: less than two-thirds of ART clinics received reports of treatment outcomes when their patients with MDR-TB were treated off-site.

In 2013, WHO recommended that Xpert MTB/RIF should be used as an initial diagnostic test in individuals with presumptive MDR-TB or HIV-associated TB [23]. Our study showed that Xpert MTB/RIF was available in about three-quarters of participating clinics. In 2012, we reported that Xpert MTB/RIF was only available on-site at 28% of ART clinics surveyed in the IeDEA consortium [24]. The increase that we observed reflects efforts to roll out Xpert MTB/RIF over the last decade, including in LMICs. Although 76% were able to diagnose MDR-TB using any Xpert MTB, fewer sites (15/29) were able to treat patients with MDR-TB. In contrast to molecular based drug resistance testing, we found that culture-based phenotypic DST was only available on-site among 35% of clinics, a marginal increase from the 26% reported in 2012 [24]. Culture is currently considered the gold standard DST method. Still, the technical challenges inherent to this method, including biosafety concerns, costly infrastructures, and slow turnover, limit its routine use at smaller ART clinics. We found that culture-based phenotypic DST was generally available at larger facilities outside of ART clinics, to which presumptive MDR-TB cases were referred to or samples were sent.

Fifty-nine per cent of clinics could test for resistance to the first-line drugs rifampicin and isoniazid and 52% to resistance of fluoroquinolones—irrespective of whether DST was performed on-site or off-site. Although DST and treatment of drug-resistant TB remains challenging in many LMICs countries, particularly at smaller clinics, it will be important to strengthen diagnostic and treatment capacities in the future. Importantly, the introduction of short-course fluoroquinolone-based treatment regimens for pan-susceptible TB, which may result in patients on failing treatment, will require DST beyond the common first-line drugs. The lack of rapid drug resistance testing to second-line drug resistance can lead to inadequate treatment and increased mortality during TB treatment [6,7]. Moreover, in a prior study, we showed that the agreement between DST performed in LMICs compared to WGS performed at a central reference laboratory was 86% for rifampicin and 65% for isoniazid, but substantially lower for second-line drugs [7]. Recent developments in genome sequencing technologies are promising, allowing point-of-care identification of resistance to first- and second-line drugs simultaneously and directly on unprocessed sputum samples [25]. Hopefully, this will allow for rapid diagnosis and effective, individualized treatment. However, next-generation sequencing methods were only available at two participating clinics. Targeted genome sequencing approaches using portable sequencers may provide promising new opportunities for rapid and reliable prediction of TB drug resistance [26].

There have been major changes in MDR-TB treatment guidelines in the last five years, including WHO’s endorsement of the injectable-containing short regimen in 2016 [18,19]. Several studies have shown high treatment success with the injectable-containing short regimen with minor modifications (80.2% to 95.5% success rates) [19,27–29]. However, one review found that patients with fluoroquinolone-resistant MDR-TB were much more likely to have an unfavorable outcome (≥50%) than those with a susceptible strain (<20%) using the injectable-containing short regimen with our without minor modifications [30]. We found that 18/29 (62%) participating clinics used the injectable-containing short regimen, and the vast majority of HCWs surveyed would use a standardized MDR-TB regimen in routine care. This was particularly evident among sites with fully integrated MDR-TB services, suggesting that access to diagnostic tools may improve guideline adherence. Overall, the clinical scenarios demonstrated reliance on a DST-guided treatment strategies. However, only a few sites had access to rapid second-line DST. Without this important test information, sites in high MDR-TB burden settings may be unable to prescribe adequate and individualized treatment regimens and struggle to achieve widespread treatment success using a standardized MDR-TB regimen [31].

A recent meta-analysis of individual patient data in MDR-TB treatment demonstrated that new or reassigned oral drugs (bedaquiline, linezolid, clofazimine, later generation fluoroquinolones and the carbapenems) were associated with increased treatment success and reduced mortality [32]. This led to new WHO guidelines, recommending the treatment of MDR-TB with only oral drugs [33]. However, we observed that access to bedaquiline and delamanid remains limited among ART clinics in LMICs. In addition improving access to new TB drugs, implementation of new MDR-TB regimens will require adherence support for MDR-TB patients to these TB drugs to ensure adequate treatment and avoid further development of drug resistance. Adherence to MDR-TB regimens remains challenging due to adverse drug reactions and drug-drug interactions, especially among PLHIV on ART [30,34]. The DOT strategy has been implemented for decades and has been considered crucial for the adherence to TB treatment [35]. However, several studies, including a systematic review, showed that more supportive interventions, such as counseling, DOT provided by trained community workers, short messaging service combined with education, monthly TB vouchers, and drug box reminders are effective for the adherence of MDR-TB patients, especially among PLHIV [35–38]. In our study, DOT was recommended during the entire MDR-TB treatment course in over 80% (24/29) of clinics.

Our study is limited in that it relies on clinicians’ reports of available services and drugs at each site rather than patient level data. Furthermore, most participating ART clinics were in urban settings and offered tertiary level care, which could have led to an overestimation of HIV-TB service integration overall. However, a notable strength of our study is the global coverage of ART clinics in LMICs with high TB burden and the detailed information collected on the on-site and off-site service availability. We have previously shown that the availability of diagnostic methods or second-line drugs do not necessarily imply that they were regularly used in routine practice [24]. We explored this gap by assessing clinical practice with the presentation of clinical scenarios.

Although most surveyed ART clinics in LMICs reported integrated HIV-TB services, less than half reported full integration of MDR-TB services and access to DST for second-line anti-TB drugs was rare. There is a continued need for increased availability of diagnostic and treatment options for MDR-TB patients and better integration of advanced MDR-TB services into the HIV care continuum in TB high-burden settings. Furthermore, there is a need for novel rapid and reliable technologies for genome-based DST such as on-site targeted genome sequencing and increased availability of newer and more effective MDR-TB drugs [26]. Repeating site-level surveys is a practical method that allows for ongoing evaluation of progressive access to such testing and treatment.

## Supplementary Material

S1 S1 Table. Questionnaire.
(DOCX)

S2 **S2 Table. Infection control measures in place at the 29 ART clinics offering TB services on- and off-site**. Abbreviations: ART, antiretroviral therapy; MDR, multidrug resistance; TB, Tuberculosis.
(DOCX)

S3 **S3 Table. Availability of TB services by the degree of integration of HIV and MDR-TB services at 29 ART clinics**. Abbreviations: DST, drug susceptibility testing; DR, drug-resistant; MDR, multidrug resistance; TB, Tuberculosis; XDR, extensive drug resistance.
(DOCX)

S4 **S4 Table. On- and off-site availability of diagnostic tests for initial TB diagnosis and diagnosis of drug-resistant TB**. Abbreviations: DST, drug susceptibility testing; LAM, urine lipoarabinomannan. * Unknown availability.
(DOCX)

S5 **S5 Table. Hypothetical clinical scenarios assessing clinical practice related to the testing and treatment of MDR-TB at ART-clinics (n = 72)**. Abbreviations: ART, antiretroviral therapy; DST, drug susceptibility testing; MDR, multidrug resistance; TB, Tuberculosis; H, isoniazid; R, rifampicin, Z, pyrazinamide; E, ethambutol.
(DOCX)

S6 S6 Table. Strobe checklist.
(DOCX)

S7 S7 Table. Participating programs and members of IeDEA.
(DOCX)

## Figures and Tables

**Table 1.** General clinic-level information, availability of TB services and cost model of care in the 29 ART clinics participating in the study, by region.

	Total (n = 29) n (%)	Asia-Pacific (n = 6) n (%)	South America (n = 3) n (%)	Africa (n = 20) n (%)
**Clinic-level information**				
**Setting**				
Urban	25 (86.2)	6 (100)	3 (100)	16 (80.0)
Rural	4 (13.8)	0	0	4 (20.0)
**Highest level of care**				
Primary	8 (27.6)	0	0	8 (40.0)
Secondary	2 (6.9)	0	0	2 (10.0)
Tertiary	19 (65.5)	6 (100)	3 (100)	10 (50.0)
**Adults and/or children care**				
Adults and children	17 (58.6)	1 (33.3)	2 (66.6)	14 (70.0)
Adults only	12 (41.4)	5 (66.7)	1 (33.3)	6 (30.0)
**Integrated HIV-TB basic services**				
Yes	25 (86.2)	6 (100)	3 (100)	16 (80.0)
No	4 (13.8)	0	0	4 (20.0)
**Availability of TB services**				
**Initial TB diagnosis**				
On-site	25 (86.2)	6 (100)	3 (100)	16 (80.0)
Off-site	4 (13.8)	0	0	4 (20.0)
**Molecular DST**				
On-site	22 (75.9)	5 (83.3)	3 (100)	14 (70.0)
Off-site	7 (21.1)	1 (16.7)	0	6 (30.0)
**Phenotypic DST**				
On-site	10 (34.5)	5 (83.3)	2 (66.7)	3 (15.0)
Off-site	19 (65.5)	1 (16.7)	1 (33.3)	17 (85.0)
**Treatment pan-susceptible TB**				
On-site	20 (69.0)	5 (83.3)	1 (33.3)	14 (70.0)
Off-site	9 (31.0)	1 (16.7)	2 (66.7)	6 (30.0)
**Treatment MDR-TB**				
On-site	15 (51.7)	5 (83.3)	1 (33.3)	9 (45.0)
Off-site	14 (48.3)	1 (16.7)	2 (66.7)	11 (55.0)
**Drug-resistant TB Integration Models**				
Full integration	14 (48.3)	5 (83.3)	1 (33.3)	8 (40.0)
Partial integration	9 (31.0)	0	2 (66.7)	7 (35.0)
Off-site only)	6 (20.7)	1 (16.7)	0	5 (25.0)
**Cost Model**				
**Initial TB diagnosis**				
Full payment by the patient	1 (3.4)	1 (16.7)	0	0
Cost sharing (partial payment by the patient)	6 (20.7)	2 (33.3)	0	4 (20.0)
Available at no cost for the patient	22 (75.9)	3 (50.0)	3 (100)	16 (80.0)
**Pan-susceptible TB treatment**				
Full payment by the patient	1 (3.4)	1 (16.7)	0	0
Cost sharing (partial payment by the patient)	1 (3.4)	1 (16.7)	0	0
Available at no cost for the patient	27 (93.1)	4 (66.7)	3 (100)	20 (100.0)
**MDR-TB treatment**				
Cost sharing (partial payment by the patient)	1 (3.4)	1 (16.7)	0	0
Available at no cost for the patient	28 (96.6)	5 (83.3)	3 (100)	20 (100)

Abbreviations: ART, antiretroviral therapy; DST, drug susceptibility testing; MDR, multidrug resistance; TB, Tuberculosis; XDR, extensive drug resistance.

https://doi.org/10.1371/journal.pgph.0000180.t001

**Table 2.** Management and treatment of MDR-TB at 29 ART clinics.

	Total (n = 29, %)	On-site (n = 15, %)	Off-site (n = 14, %)	p-value
**Treatment in line with National TB Program**				0.37
Strictly in line with National TB Program	27 (93.1)	13 (86.7)	14 (100)	
Somewhat modified	1 (3.4)	1 (6.7)	0	
Individualised MDR-TB treatment	1 (3.4)	1 (6.7)	0	
**Directly observed treatment**				
During initiation phase only	5 (17.2)	0	5 (35.7)	0.017
During the whole duration of treatment	24 (82.8)	15 (100)	9 (64.3)	
**Clinical visits intensive phase**				0.75
Daily	8 (27.6)	5 (33.3)	3 (21.4)	
Weekly	9 (31.0)	4 (26.7)	5 (35.7)	
Monthly	12 (41.4)	6 (40.0)	6 (42.9)	
**Clinical visits continuation phase**				0.81
Daily	2 (6.9)	1 (6.7)	1 (7.1)	
Weekly	2 (6.9)	2 (13.3)	0	
Monthly	22 (75.9)	11 (73.3)	11 (78.6)	
Every third month	3 (10.3)	1 (6–7)	2 (14.3)	
**Type of directly observed treatment**				0.79
Self-administered	5 (17.2)	4 (26.7)	1 (7.1)	
Health facility based	11 37.9)	5 (33.3)	6 (42.9)	
Health and self-administered	11 (37.9)	6 (40.0)	5 (35.7)	
Unknown	2 (6.9)	0	2 (14.3)	
**MDR-TB regimens**				
Individualised according to the resistance profile	13 (44.8)	10 (66.7)	3 (21.4)	0.062
Standardised	11 (37.9)	3 (30.0)	8 (57.1)	
Standardised or according to the resistance profile	5 (17.2)	2 (13.3)	3 (21.4)	
**Use of injectable-containing short regimen “Bangladesh”**				1.0
Yes	18 (62.1)	9 (60.0)	9 (64.3)	
No	9 (31.0)	5 (33.3)	4 (28.6)	
Unknown	2 (6.9)	1 (6.7)	1 (7.1)	
**Availability of new drugs**				
**Bedaquiline**				0.02
Yes, always	5 (17.2)	4 (26.7)	1 (7.1)	
Upon request	7 (24.1)	6 (40.0)	1 (7.1)	
No	17 (58.6)	5 (33.3)	12 (85.7)	
**Delamanid**				0.08
Yes, always	3 (10.3)	3 (20.0)	0	
Upon request	7 (24.1)	5 (33.3)	2 (14.3)	
No	19 (65.5)	7 (46.7)	12 (85.7)	

Abbreviations: MDR, multidrug resistance; TB, Tuberculosis.

https://doi.org/10.1371/journal.pgph.0000180.t002

## Data Availability

Complete data for this study cannot be posted in a supplemental file or a public repository because of legal and ethical restrictions. The Principles of Collaboration of this multi-national consortium and the regulatory requirements of the different countries’ IRBs require the submission and approval of individual project concept sheets that describe the planned analyses. Specifically, while the data held by the IeDEA consortium may be available to other investigators, proposed use must be based on a concept note that is approved by the Response to editorial office 2 regional Steering Groups and the IeDEA Executive Committee (Chairperson; Annette Sohn, MD; annette.sohn@treatasia.org).
