# Supplementary material for: Integrating services for HIV and multidrug-resistant tuberculosis: A global cross-sectional survey among ART clinics in low- and middle-income countries
Source: PLOS Glob Public Health. 2022 Mar 1;2(3):e0000180. doi: 10.1371/journal.pgph.0000180 (PMC9910322; doi:10.1371/journal.pgph.0000180)
Supplement: S1 Table — (DOCX) [file pgph.0000180.s001.docx]

**S1 Table: Questionnaire**

| **MDR Basic Information**  **1. Basic information about your ART facility**  IeDEA Data Manager may complete section 1 and may help collect responses but for the remainder of the questionnaire. The remainder of the questionnaire should be complete from the perspective  of a health care worker at the ART facility who provides direct care to patients. Section 1 only needs to be submitted once for each facility. | | |
| --- | --- | --- |
| 1.1. IeDEA Region |            | Asia/Pacific  Caribbean, Central and South America Central Africa  East Africa  Southern Africa West Africa |
| 1.2. Cohort or Center ID (One answer only) |  | …………………………………… |
| 1.3. Setting |      | Urban  Peri-urban (immediately adjoining urban areas) Rural |
| 1.4. Level of care |        | Primary (health care center or facility) Secondary (district or provincial hospital) Tertiary (teaching or referral hospital, at district or provincial level)  Other (specify) |
| If other please specify: |  | |
| 1.5. Are adults (>16 yrs) and children treated at your ART facility? |      | Adults only (>15 yrs) Children only (<15 yrs)  Both adults and children |
| 1.6. Size of HIV cohort: total number of adult cases (>16 yrs) actively being followed up on at time  of survey completion | …………………………………………………………………………………… | |
| 1.7. Size of HIV cohort: total number of child cases (<16 yrs) actively being followed up on at time  of survey completion | …………………………………………………………………………………… | |
| 1.8. Number of newly detected adult TB cases (including drug-susceptible, MDR-TB, XDR-TB, pulmonary and extrapulmonary TB) per year at  your ART facility | Number: …………………………… Year: ……………………………..  (per year (indicate date of most recent data)) N/A (suspected TB cases are referred elsewhere) | |
| 1.9. Number of newly detected child TB cases (including drug-susceptible, MDR-TB, XDR-TB, pulmonary and extrapulmonary TB) per year at  your ART facility | Number: …………………………… Year: ……………………………..  (per year (indicate date of most recent data))  N/A (suspected TB cases are referred elsewhere) | |

| 1.10. Number of newly detected adult MDR-TB cases (including pulmonary and extrapulmonary TB) per year at your ART facilty. | Number: …………………………… Year: ……………………………..  (per year (indicate date of most recent data)) N/A (suspected MDR-TB cases are referred  elsewhere) | |
| --- | --- | --- |
| 1.11. Number of newly detected child MDR-TB cases (including pulmonary and extrapulmonary TB) per year at your ART facility | Number: …………………………… Year: ……………………………..  (per year (indicate date of most recent data)) N/A (suspected MDR-TB cases are referred  elsewhere) | |
| 1.12. Number of newly detected adult XDR-TB cases (including pulmonary and extrapulmonary TB) per year at your ART facility | Number: …………………………… Year: ……………………………..  (per year (indicate date of most recent data))  N/A (suspected XDR-TB cases are referred elsewhere) | |
| 1.13. Number of newly detected children XDR-TB cases (all cases) per year at your ART facility | Number: …………………………… Year: ……………………………..  (per year (indicate date of most recent data))  N/A (suspected XDR-TB cases are referred elsewhere) | |
| 1.14. Source of TB drug supply at your treatment program |                | National TB program / government Green Light Committee  Private pharmacy Global Fund PEPFAR  Bill Gates Foundation Other  Unknown |
| **2. Basic Information about Respondent**  Respondent must be a health care worker at the ART facility who provides direct care to patients. | | |
| 2.1 If someone else, indicate their profession. (only one answer)  (If ‘clinical officer, nurse, other or I don’t know`, SKIP to next section) |          | Medical doctor Clinical officer Nurse  Other, please specify: …………………………………………  I don’t know |
| 2.2.1 Please indicate their medical specialty, if applicable. (only one answer) |                | Infectious diseases Pulmonology General Medicine Pediatrics general  Pediatrics infectious diseases Pediatrics pulmonology  Other, please specify: ……………………………………..  N/A |
| **3. Diagnostics and Treatment Costs** | | |
| 3.1. Does your country have a health insurance system in place? |      | Yes No  Unknown |
| 3.2. Which of the following best describes your cost model for ....  (if a health insurance system is in place, indicate the cost model which applies to most of your patients) | | |

|  | Full payment by the patient | Cost sharing  (partial payment by the patient) | | Available at  no cost for the patient | Other | Unknown |
| --- | --- | --- | --- | --- | --- | --- |
| HIV diagnostics HIV treatment  Initial TB diagnostics  Pan-susceptible TB treatment Drug resistance testing  MDR-TB treatment |            |            | |            |            |            |
| If other please specify: HIV diagnostics  HIV treatment Initial TB diagnostics  Pan-susceptible TB treatment Drug resistance testing  MDR-TB treatment | ………………………………………………………………………………..  ………………………………………………………………………………..  ………………………………………………………………………………..  ………………………………………………………………………………..  ………………………………………………………………………………..  ……………………………………………………………………………….. | | | | | |
| 3.3. Currency used for the following questions (If ‘USD, SKIP to next section ) | | |  USD   Other currency | | | |
| 3.3.1 If other currency, name of the currency: | | | …………………………………………………………………… | | | |
| 3.3.2 Exchange rate of currency used | | | ……………………………………………………………………  (1 USD = ? ). | | | |
| **4. Cost to Patient of Individual items**  Please note: Enter "0" if test is for free to the Patient and "-99" if it is unknown. | | | | | | |
| 4.1 Cost of mycobacterial culture (initial culture), per culture | | | ………………………………………………………………………  (Enter "0" if test is for free to the patient. Enter "-99" if it is  unknown.) | | | |
| 4.2 Xpert MTB/RIF, per test assay | | | ………………………………………………………………………  (Enter "0" if test is for free to the patient. Enter "-99" if it is  unknown.) | | | |
| 4.3 Xpert Omni, per test assay | | | ………………………………………………………………………  (Enter "0" if test is for free to the patient. Enter "-99" if it is  unknown.) | | | |
| 4.4 Xpert Ultra, per test assay | | | ………………………………………………………………………  (Enter "0" if test is for free to the patient. Enter "-99" if it is  unknown.) | | | |
| 4.5 Xpert XDR, per test assay | | | ………………………………………………………………………  (Enter "0" if test is for free to the patient. Enter "-99" if it is  unknown.) | | | |
| 4.6 HAIN MTBDRplus, per test assay | | | ………………………………………………………………………  (Enter "0" if test is for free to the patient. Enter "-99" if it is  unknown.) | | | |
| 4.7 HAIN MTBDRsl, per test assay | | | ………………………………………………………………………  (Enter "0" if test is for free to the patient. Enter "-99" if it is  unknown.) | | | |
| 4.8 First line anti-TB drugs, per day | | | ………………………………………………………………………  (Enter "0" if test is for free to the patient. Enter "-99" if it is  unknown.) | | | |

| 4.9 Second line anti-TB drugs (oral), per day | ………………………………………………………………………  (Enter "0" if test is for free to the patient. Enter "-99" if it is  unknown.) |
| --- | --- |
| 4.10 Second line anti-TB drugs (fluoroquinolones) , per day | ………………………………………………………………………  (Enter "0" if test is for free to the patient. Enter "-99" if it is  unknown.) |
| 4.11 Second line anti-TB drugs (injectable), per day | ………………………………………………………………………  (Enter "0" if test is for free to the patient. Enter "-99" if it is  unknown.) |
| 1.12 Third line anti-TB drugs, per day | ………………………………………………………………………  (Enter "0" if test is for free to the patient. Enter "-99" if it is  unknown.) |

| **MDR Adults**  **1. Basic information about your ART facility**  Respondent must be a health care worker at the ART facility who provides direct care to patients. | | |
| --- | --- | --- |
| 1.1. IeDEA Region |            | Asia/Pacific  Caribbean, Central and South America Central Africa  East Africa  Southern Africa West Africa |
| 1.2. Cohort or Center ID (one answer only) |  | ………………………………………… |
| **2. Basic Information about Respondent**  Respondent must be a health care worker at the ART facility who provides direct care to patients. | | |
| 2.1. Profession of person completing this survey |  | Medical doctor |
| (one answer only) |  | Clinical officer |
|  |  | Nurse |
| (If ‘clinical officer or nurse`, SKIP to Q 2.2) |  | Other, please specify: ………………………………………… |
| 2.1.1. Please indicate your medical specialty, if applicable.  (one answer only) |                | Infectious diseases Pulmonology General Medicine Pediatrics general  Pediatrics infectious diseases Pediatrics pulmology  Other, please specify: …………………………………………  N/A |
| 2.2. How many years of work experience do you have in HIV care?  (one answer only) |      | Less than 1 year Between 1 and 5 years More than 5 years |
| 2.3. How many years of work experience do you have at this ART facility?  (one answer only) |      | Less than 1 year Between 1 and 5 years  More than 5 years |
| **3. Case Definition** | | |
| 3.1. Pan-susceptible TB: How is TB case defined at |  | Bacteriologically confirmed TB case |
| your ART facility? (one answer only) |  | Clinically diagnosed TB case |
|  |  | Both of the above |
|  |  | None of the above |

|  |  | I don’t know |
| --- | --- | --- |
| 3.2. MDR-TB: What is the definition of multidrug- resistant TB at your ART facility? (only one answer) |          | Resistance to rifampicin only Resistance to isoniazid only  Resistance to any first-line anti-TB medications  Resistance to rifampicin and isoniazid Resistance to any two first line anti-TB |
|  |  | medications |
|  |    | None of the above I don’t know |
| 3.3. XDR-TB: What is the WHO definition of extensively drug-resistant TB? (only one answer) |    | Resistance to any three anti-TB medications  Resistance to rifampicin, plus any second-line anti-TB medication |
|  |  | Resistance to rifampicin, isoniazid, plus any second-line anti-TB medication |
|  |  | Resistance to rifampicin, isoniazid, plus any fluoroquinolone and at least one injectable  second-line drug |
|  |    | None of the above I don’t know |
| 3.4. How are MDR-TB cases confirmed at your ART facility? (only one answer) |      | By testing for rifampicin resistance only By testing for isoniazid resistance only  By testing for at least isoniazid and rifampicin |
|  |  | resistance |
|  |      | By testing for any resistance to anti-TB drugs Through clinical observation only  We refer MDR-TB suspects out to another |
|  |  | location for drug-resistance testing |
|  |    | I don’t know  Other, please specify: ………………………………………… |

| **4. Management of TB and MDR-TB at ART facilities** | | | | |
| --- | --- | --- | --- | --- |
|  | **For initial TB diagnosis** | | **For drug susceptibility testing** | |
| **Diagnostics** |  | |  | |
| 4.1. You suspect that a patient at your ART facility has TB. Are TB diagnostic tests done TB service at same location as your ART facility |      | Yes No  I don’t know |      | Yes No  I don’t know |
| (If ‘yes’, SKIP to Q 4.3) |  |  |  |  |
| 4.1.1. Where are the TB diagnostic tests performed? |  | At TB service at same location as your ART |  | At TB service at same location as your ART |
| (only one answer) |        | facility  At TB service at a different location No tests are performed  Other, please specify: ……………………………………….  I don’t know |        | facility  At TB service at a different location No tests are performed  Other, please specify: ………………………………………  I don’t know |
| 4.1.2. Is the TB suspect referred out of your ART facility to receive diagnostics or does your facility just send out their samples? |      | Patient referred out Samples sent  I don’t know |      | Patient referred out Samples sent  I don’t know |
| 4.1.3. How far away from your ART facility is the location where TB diagnostic tests are performed? (only one answer) |        | Less than 20 km Between 20 and 40 km More than 40 km  I don’t know |        | Less than 20 km Between 20 and 40 km More than 40 km  I don’t know |
| 4.2. Who prescribes the TB diagnostic tests? (only one answer)  (If ‘myself skip to Q4.3.) |    | Myself Someone else |    | Myself Someone else |
| 4.2.1. If someone else, indicate their profession. |        | Medical doctor Clinical officer Nurse  I don’t know |        | Medical doctor Clinical officer Nurse  I don’t know |
| (only one answer) |  |  |  |  |
| (If ‘clinical officer, nurse or I don’t know`, |  |  |  |  |
| SKIP to Q 4.3) |  |  |  |  |
| 4.2.2. Please indicate their medical specialty, if applicable. (only one answer) |          | Infectious diseases Pulmonology General Medicine Pediatrics general  Pediatrics infectious diseases |          | Infectious diseases Pulmonology General Medicine Pediatrics general  Pediatrics infectious diseases |

|  |      | Pediatrics pulmonology  Other, please specify: ……………………………………..  N/A |      | Pediatrics pulmonology  Other, please specify: ………………………………………  N/A |
| --- | --- | --- | --- | --- |
| 4.3. After the TB diagnostics are completed, do you receive or have access to the results? (only one answer) (If ‘never’ SKIP to 4.4) |      | Always Sometimes  Never |      | Always Sometimes  Never |
| 4.3.1. How is the TB diagnostic result shared? |  | In the patients file, which remains with the |  | In the patients file, which remains with the |
| (multiple answer) |    | patient  In the patients file, which remains at the facility In the patients file, which remains at a different |    | patient  In the patients file, which remains at the facility In the patients file, which remains at a different |
|  |          | location  In an electronic a database Verbally by phone Verbally face-to-face  Other, please specify: ……………………………………….  I don’t know |          | location  In an electronic a database Verbally by phone Verbally face-to-face  Other, please specify: ………………………………………  I don’t know |
| **Treatment** |  | |  | |
| 4.4. Are TB patients treated for TB at the TB service at same location as your ART facility?  (If ‘yes’, SKIP to Q4.5) |      | Yes No  I don’t know |      | Yes No  I don’t know |
| 4.4.1. Where are TB patients referred to for |  | At TB service at same location as your ART |  | At TB service at same location as your ART |
| treatment? (only one answer) |        | facility  At TB service at a different location No tests are performed  Other, please specify: ……………………………………….  I don’t know |        | facility  At TB service at a different location No tests are performed  Other, please specify: ………………………………………  I don’t know |
| 4.4.2. How far away from your ART facility is the location were TB patients are referred for treatment? (only one answer) |        | Less than 20 km Between 20 and 40 km More than 40 km  I don’t know |        | Less than 20 km Between 20 and 40 km More than 40 km  I don’t know |
| 4.5. Who prescribes TB treatment at your ART facility? (only one answer)  (If ‘myself or N/A’, SKIP to Q4.6 . |      | Myself Someone else  N/A – TB patients never treated at our ART  facility |      | Myself Someone else  N/A – TB patients never treated at our ART  facility |
| 4.5.1. If someone else, please indicate their profession.(only one answer) |    | Medical doctor Clinical officer |    | Medical doctor Clinical officer |

| (If ‘clinical officer, nurse or I don’t know’,  SKIP to Q 4.6) |  Nurse   I don’t know |  Nurse   I don’t know |
| --- | --- | --- |
| 4.5.2. Please indicate their medical specialty, if applicable. |  Infectious diseases   Pulmonology   General Medicine   Pediatrics general   Pediatrics infectious diseases   Pediatrics pulmonology   Other, please specify: ……………………………………….   N/A |  Infectious diseases   Pulmonology   General Medicine   Pediatrics general   Pediatrics infectious diseases   Pediatrics pulmonology   Other, please specify: ………………………………………   N/A |
| 4.6. If a TB patient is referred elsewhere for treatment, does your ART facility share information with them on the patient’s ARV regimen? (only one answer)  If ‘Never’, I don’t know or N/A’ SKIP to next section 5. |  Always   Sometimes   Never   I don’t know   N/A – TB patients always treated at our ART facility |  Always   Sometimes   Never   I don’t know   N/A – TB patients always treated at our ART facility |
| 4.6.1. I information is sometimes or always shared, how is that information shared? (multiple answers) |  In the patients file, which remains with the patient   In the patients file, which remains at the facility   In the patients file, which remains at a different location   In an electronic a database   Referral letter   Verbally by phone   Verbally face-to-face   Other, please specify: ……………………………………….   I don’t know |  In the patients file, which remains with the patient   In the patients file, which remains at the facility   In the patients file, which remains at a different location   In an electronic a database   Referral letter   Verbally by phone   Verbally face-to-face   Other, pleases specify: …………………………………….   I don’t know |
| 4.7. If a TB patient is referred elsewhere for treatment, do you receive or have access to progress reports about the patient? (only one answer)  (If ‘Never or N/A’ SKIP to Q4.8) |  Always   Sometimes   Never   N/A – TB patients always treated at our ART facility |  Always   Sometimes   Never   N/A – TB patients always treated at our ART facility |
| 4.7.1. If you receive a progress report sometimes or always, how frequently do you receive a progress report about the referred TB patient? (only one answer) |  Weekly   Monthly   Every 3 months   Every 6 months |  Weekly   Monthly   3 monthly   6 monthly |

|  |  Yearly   Other, please specify: ……………………………………….   I don’t know |  Yearly   Other, please specify: ………………………………………   I don’t know |
| --- | --- | --- |
| 4.8. If a TB patient is referred elsewhere for treatment, do you receive a final report on the referred patient’s? (only one answer) |  Always   Sometimes   Never   N/A – TB patients always treated at our ART facility |  Always   Sometimes   Never   N/A – TB patients always treated at our ART facility |
| 4.8.1. If a final report is sometimes or always received, how is the final report between your ART facility and where the patient is treated shared? (multiple answers) |  In the patients file, which remains with the patient   In the patients file, which remains at the facility   In the patients file, which remains at a different location   In an electronic a database   Verbally by phone   Verbally face-to-face   Other, please specify: ………………………………………   I don’t know |  In the patients file, which remains with the patient   In the patients file, which remains at the facility   In the patients file, which remains at a different location   In an electronic a database   Verbally by phone   Verbally face-to-face   Other, please specify: ……………………………………   I don’t know |

| **5. Availability of diagnostic tools for TB drug resistance** | | | | | | | | | |
| --- | --- | --- | --- | --- | --- | --- | --- | --- | --- |
| 5.1 Please select the diagnostic tests which are available to diagnose any form of TB (including to test for drug  resistance) (only one answer per test) | | | | | | | | | |
|  | | Available at TB service at same location as  your ART facility | | Available at TB service at different  location | | | Not available | | I don’t know |
| Chest X-ray  Sputum smear microscopy Tuberculin skin testing Interferon-gamma release tests Mycobacterial culture  Xpert MTB/RIF Xpert Ultra Xpert XDR Xpert Omni  HAIN MTBDRplus HAIN MTBDRsl  Urine LAM  Other: please specify:  ……………………………………………….. | |                        | |                        | | |                        | |                        |
| 5.2 If test are available at different location, how far away from your ART facility (including tests for drug resistance testing, only one answer per test) | | | | | | | | | |
|  | | Less than 20 km | | Between 20 and 40  km | | | More than 40  km | | I don’t know |
| Chest X-ray  Sputum smear microscopy Tuberculin skin testing Interferon-gamma release tests Mycobacterial culture  Xpert MTB/RIF Xpert Ultra Xpert XDR Xpert Omni  HAIN MTBDRplus HAIN MTBDRsl  Urine LAM  Other: please specify:  ……………………………………………….. | |                        | |                        | | |                        | |                        |
| 5.3 Please indicate how each test is used at our facility (only one answer per test) | | | | | | | | | |
|  | All patients for screening | | Only patients clinically suspected of drug resistance | | Only patients failing TB treatment | Patients clinically suspected of drug resistance and patients failing TB  treatment | | Other | I don‘t know |

| Chest X-ray |                        |                        | |                        |                        |                        |                        |
| --- | --- | --- | --- | --- | --- | --- | --- |
| Sputum smear microscopy |  |  |  |  |  |  |  |
| Tuberculin skin testing |  |  |  |  |  |  |  |
| Interferon-gamma release tests |  |  |  |  |  |  |  |
| Mycobacterial culture |  |  |  |  |  |  |  |
| Xpert MTB/RIF |  |  |  |  |  |  |  |
| Xpert Ultra |  |  |  |  |  |  |  |
| Xpert XDR |  |  |  |  |  |  |  |
| Xpert Omni |  |  |  |  |  |  |  |
| HAIN MTBDRplus |  |  |  |  |  |  |  |
| HAIN MTBDRsl |  |  |  |  |  |  |  |
| Urine LAM |  |  |  |  |  |  |  |
| 5.3.1 If other please specify: | | | | | | | |
| Chest X-ray | **…………………………………………………………** | | | | | | |
| Sputum smear microscopy | **…………………………………………………………** | | | | | | |
| Tuberculin skin testing | **…………………………………………………………** | | | | | | |
| Interferon-gamma release tests | **…………………………………………………………** | | | | | | |
| Mycobacterial culture | **…………………………………………………………** | | | | | | |
| Xpert MTB/RIF | **…………………………………………………………** | | | | | | |
| Xpert Ultra | **…………………………………………………………** | | | | | | |
| Xpert XDR | **…………………………………………………………** | | | | | | |
| Xpert Omni | **…………………………………………………………** | | | | | | |
| HAIN MTBDRplus | **…………………………………………………………** | | | | | | |
| HAIN MTBDRsl | **…………………………………………………………** | | | | | | |
| Urine LAM | **…………………………………………………………** | | | | | | |
| *Mycobacterial culture* | | | | | | | |
| 5.4 If culture is available at TB service at same location as | | |              | MGIT 960 liquid culture system BACTEC 460 liquid culture system  Solid media (Löwenstein-Jensen, 7H10, 7H11, etc.) BACTEC and Solid media  Other, please specify: ……….………………………………….  I don’t know N/A | | | |
| your ART facility or at different location. Type of culture | | |  |  |  |  |  |
| system (multiple answer) | | |  |  |  |  |  |
| (If ‘N/A or I don’t know’ SKIP to Q5.6) | | |  |  |  |  |  |
| 5.5 For which drugs can phenotypic drug susceptibility testing be performed? (check all that apply) | | |                                    | Amikacin Amoxicillin/clavulanic acid Bedaquiline  Delamanid Ciprofloxacin Clarithromycin Clofazimin Capreomycin Cycloserine Ethambutol Ethionamide Gatifloxacin Imipenem/cilastatin Isoniazid Kanamycin Levofloxacin  Linezolid Meropenem | | | |

|  | | | | |                        | Moxifloxacin Ofloxacin  Para-aminosalicylic acid Para-aminosalicylate sodium Protionamide  Pyrazinamide Rifabutin Rifampicin Streptomycin Terizidone  Other, please specify: ……………………………………………  None | | | | |
| --- | --- | --- | --- | --- | --- | --- | --- | --- | --- | --- |
| *GeneXpert generations or Xpert* | | | | | | | | | | |
| 5.6 If culture is available at TB service at same location as your ART facility or at different location. Have you any experienced cartridge stock outs in the last 12 months at your ART facility. (one answer per test)  If ‘no’ or ‘I don’t know’ SKIP to Q5.7 | | | | | | | | | | |
|  | | Yes | | | No | | | | I don’t know | |
| Xpert MTB/RIF Xpert Ultra Xpert XDR Xpert Omni | |        | | |        | | | |        | |
| 5.6.1 If yes, how frequently have you experienced cartridge stock out in the last 12 months? (one answer per test) | | | | | | | | | | |
|  | Weekly | | Monthly | Every 3 months | | | Every 6 months | Only once | | I don’t know |
| Xpert MTB/RIF Xpert Ultra Xpert XDR Xpert Omni |        | |        |        | | |        |        | |        |
| **Sequencing methods** | | | | | | | | | | |
| 5.7 Does your facility has access to sequencing methods? (only one answer)  If ‘no or I don’t know’ SKIP to next section 6. | | | | |      | Yes No  I don’t know | | | | |
| 5.7.1 If yes, to which sequencing method does your ART facility has access? (only one answer) | | | | |        | Pyrosequencing Nanopore  Whole genome sequencing  Other, please specify: …………………………………………… | | | | |
| **6. MDR diagnostic algorithm at your ART facility (NOT at the referral facility/hospital)**  We kindly ask you to upload your national drug-resistant TB guidelines for TB diagnostics and for the treatment. If unavailable, we would like to gather some information about your diagnostic algorithm. | | | | | | | | | | |
| 6.1. Please upload your national guidelines for the  diagnosis of drug-resistant TB | | | | |  | | | | | |
| 6.2. According to the diagnostic algorithm in use at your ART facility, which drug-resistance test do you use when a drug-resistant TB case is suspected? (multiple answer)  If ‘none’ SKIP to next section. | | | | |              | Xpert MTB/RIF Xpert Ultra Xpert XDR Xpert Omni  HAIN MTBDRplus HAIN MTBDRsl  Culture for phenotypic drug resistance testing | | | | |

|  |  | Culture for genotyping or whole genome |
| --- | --- | --- |
|  |  | sequencing |
|  |  | Other, please specify: …………………………………………… |
|  |  | None |
|  |  | I don’t know |
| 6.3. If your ART facility performs drug-resistance testing, for which drugs are you testing? (multiple answer) |                                                            | Amikacin Amoxicillin/clavulanic acid Bedaquiline  Delamanid Ciprofloxacin Clarithromycin Clofazimin Capreomycin Cycloserine Ethambutol Ethionamide Gatifloxacin Imipenem/cilastatin Isoniazid Kanamycin Levofloxacin Linezolid Meropenem Moxifloxacin Ofloxacin  Para-aminosalicylic acid Para-aminosalicylate sodium Protionamide  Pyrazinamide Rifabutin Rifampicin Streptomycin Terizidone  Other, please specify: ……………………………………………  None |
| 6.1. Do you perform a second drug-resistance test to confirm the result? (only one answer)  If ‘yes (same method, no or I don’t know’ SKIP to next section 7. |        | Yes (same method used twice)  Yes (confirmation with another method) No  I don’t know |
| 6.4.1. If another method, which method is used as a confirmatory test? (multiple answer) |                    | Xpert MTB/RIF Xpert Ultra Xpert XDR Xpert Omni  HAIN MTBDRplus HAIN MTBDRsl  Culture for phenotypic DST  Culture for whole genome sequencing  Other, please specify: ……………………………………………  I don’t know |
| 6.4. Do you perform a third drug-resistance test? (only one answer) |    | Yes  No |

| If ‘no or I don’t know’ SKIP to next section 7. |  | I don’t know |
| --- | --- | --- |
| 6.5.1 If yes, which drug-resistance test is used for the third test? (multiple answer) |                    | Xpert MTB/RIF Xpert Ultra Xpert XDR Xpert Omni  HAIN MTBDRplus HAIN MTBDRsl  Culture for phenotypic DST  Culture for whole genome sequencing  Other, please specify: ……………………………………………  I don’t know |
| **7. Treatment of MDR-TB patients at your ART facility** | | |
| 7.0. Please upload your national guidelines for the  treatment of drug-resistant TB |  | |
| 7.1. Are your treatment regimens in line with recommendations from the National TB Program? (only one answer) |        | Strictly in line with National TB Program Somewhat modified  Individualized MDR-TB treatment  Unknown |
| 7.2. Is directly observed treatment (DOT) recommended |  | Never |
| for MDR-TB? (only one answer) |  | During initiation phase only |
|  |  | During the whole duration of treatment |
|  |  | Unknown |
| 7.3. If used, type of directly observed treatment for |  | Self-administered (include home and community |
| MDR-TB? (only one answer) |    | based)  Health facility-based  From health facility-based during intensive phase |
|  |  | to self-admistered  From self-admistered during intensive phase to |
|  |    | health facility-based  Other, please specify: ……………………………………………  Unknown |
| 7.4. How frequently are MDR-TB patients seen by the clinician during the intensive phase (responsible clinician for TB treatment)?(only one answer) |                  | Daily Weekly Monthly  Every 2^nd^ month Every 3^rd^ month Half yearly Irregularly  Other, please specify:………………………………………….  Unknown |
| 7.5. How frequently is the MDR-TB patient seen by the clinician during the continuous phase (responsible clinician for TB treatment)? (only one answer) |                  | Daily Weekly Monthly  Every 2^nd^ month Every 3^th^ month Half yearly Irregularly  Other, please specify:………………………………………….  Unknown |

| 7.6. Are MDR-TB regimens individualized at your ART facility? (only one answer) |  Yes, according to the resistance profile   No   Some, according to the resistance profile   Unknown |
| --- | --- |
| 7.7. Do you use the the “Bangladesh regimen” at your ART facility to treat HIV patients co-infected with MDR-TB:  Bangladesh regimen : 4-6 months with KmMfxPtoCfzZEH^high-dose^, 5 months with MfxCfzZE)”? (only one answer) |  Yes   No   Some   Unknown |
| 7.8. Which anti-TB drugs are available at your ART facility? (multiple answer) |  Amikacin   Amoxicillin/clavulanic acid   Bedaquiline   Delamanid   Ciprofloxacin   Clarithromycin   Clofazimin   Capreomycin   Cycloserine   Ethambutol   Ethionamide   Gatifloxacin   Imipenem/cilastatin   Isoniazid   Kanamycin   Levofloxacin   Linezolid   Meropenem   Moxifloxacin   Ofloxacin   Para-aminosalicylic acid   Para-aminosalicylate sodium   Protionamide   Pyrazinamide   Rifabutin   Rifampicin   Streptomycin   Terizidone   Other, please specify: …………………………………………..   None |
| 7.9. Do you have experienced shortages of anti-TB drugs at your ART facility?  If ‘no or unknown’ SKIP to Q7.10. |  Yes   No   Unknown |
| 7.9.1 If yes, please tick any of the drugs for which a shortage has ever been experienced. (multiple answer) |  Amikacin   Amoxicillin/clavulanic acid   Bedaquiline   Delamanid   Ciprofloxacin   Clarithromycin   Clofazimin   Capreomycin |

|  | | | | |                                            | Cycloserine Ethambutol Ethionamide Gatifloxacin Imipenem/cilastatin Isoniazid Kanamycin Levofloxacin Linezolid Meropenem Moxifloxacin Ofloxacin  Para-aminosalicylic acid Para-aminosalicylate sodium Protionamide  Pyrazinamide Rifabutin Rifampicin Streptomycin Terizidone  Other, please specify: ……………………………………………  None | | | | |
| --- | --- | --- | --- | --- | --- | --- | --- | --- | --- | --- |
| 7.9.2 If yes, how frequently are shortage occuing? (only one answer) | | | | |            | Weekly Monthly Three monthly Half yearly  Other, please specify: ………………………………………….  Unknown | | | | |
| 7.9.3 If yes, what is the most common duration of a shortage? (only one answer) | | | | |        | Less than 1 week  Between 1 and less than 2 weeks  Between 4 and less than 4 weeks More than 4 weeks | | | | |
| If yes, when there are shortages of anti-TB drugs, how do you manage patients? (multiple answer) | | | | |              | Treatment is interrupted  Refer the patient to another MDR-TB treatment facility/hospital  Replace the drug with another first or second line drug  Contact a nearby facility/hospital to borrow drugs Contact the National TB Program and request shipment  Other, please specify: ……………………………………………  Unknown | | | | |
| 7.10 Are the following two anti-TB drugs available at your ART facility? (only one answer per drug) | | | | | | | | | | |
|  | | Yes, always | | Upon request | | | No | | Unknown | |
| Bedaquiline Delamanid | |    | |    | | |    | |    | |
| 7.10.1 If upon request or always, for whom are the following anti-TB drugs used? (multiple answer) | | | | | | | | | | |
|  | Pan-susceptible  TB patients | | MDR-TB patients | | XDR-TB patients | | | Other | | Unknown |
| Bedaquiline Delamanid |    | |    | |    | | |    | |    |

| **8. Management of MDR-TB treatment side effects at your ART facility** | | |
| --- | --- | --- |
| 8.1 Are side effects due to the MDR regimen recorded at |  | Yes, at TB service at same location as your ART |
| your ART facility? (only one answer) |        | facility  Yes, at TB service at different location No  Other, please specify: …………………………………………..  Unknown |
| 8.2 How are side effects due to the MDR regimen captured, if not at the ART facility? (multiple answer) |      | In the patients file, which remains with the patient In the patients file, which remains at the facility  In the patients file, which remains at a different |
|  |            | location  In an electronic a database Referral letter  Verbally by phone Verbally face-to-face  Other, please specify: ………………………………………….  I don’t know |
| 8.3 Are side effects due to the MDR regimen managed at |  | Always |
| your ART facility? (only one answer) |  | Sometimes |
|  |  | Never |
|  |  | Unknown |
| 8.4 Who captures the side effects?(only one answer) (If ‘myself’, SKIP to next section 9) |    | Myself  Someone else |
| 8.4.1 Please indicate their profession. |        | Medical doctor Clinical officer Nurse  I don’t know |
| (only one answer) |  |  |
| (If ‘clinical officer, nurse or I don’t know’, SKIP to next |  |  |
| section 9) |  |  |
| 8.4.2 If medical doctor, their medical specialty, if applicable |                | Infectious diseases Pulmonology General Medicine Pediatrics general  Pediatrics infectious diseases Pediatrics pulmonology Other  N/A |
| **9. XDR-TB management** | | |
| 9.1. Are XDR-TB patients treated at your ART facility? (If ‘no or unknown’, SKIP to next section 10) |      | Yes No  Unknown |
| 9.2. Are your XDR-TB treatment regimens in line with |        | Strictly in line with the National TB Program Somewhat modified  Individualized XDR-TB treatment  Unknown |
| recommendations from the National TB Program? |  |  |
| (only one answer) |  |  |
| 9.3. Are the regimens for the XDR-TB patients standardized? (only one answer)  (If ‘no or unknwon’, SKIP to next section 10) |      | Yes No  Unknown |

| 9.4. If yes: What is the standardized XDR-TB regimen? | ………………………………………………………………………………….. Please indicate the drugs and duration of the regimen. Please indicate in the following format: “2HRZE, 4RH” | |
| --- | --- | --- |
| **10.Infection control measures for any TB patients at your ART program** | | |
| 10.1 Separate waiting rooms for MDR patients (only one answer) |      | Yes No  Unknown |
| 10.2 Separate visit hours for MDR patients |      | Yes No  Unknown |
| 10.3 Natural air exchange through windows (only one answer) |    | Optimized natural ventilation (e.g.  airflow optimized by size of windows and location |
|  |  | on opposing walls) |
|  |      | Natural ventilation, but not optimized No natural ventilation  Unknown |
| 10.4 Protection Prevention of medical staff working with |  | Regular TB symptom screening for (coughing, |
| any TB patients or TB suspects (multiple answers  possible) |    | sweats, fever, etc.)  Regular screening by chest X-ray  Regular TB screening by sputum smear regardless |
|  |  | of symptoms  Regular TB screening by culture regardless of |
|  |  | symptoms  Regular TB screening by molecular tests regardless |
|  |  | of symptoms  Wearing masks if in close contact to any TB |
|  |      | patients |
|  |  | Other, please specify: …………………………………………  No specific protection offered to medical staff Unknown |
| **11.Intensified tuberculosis case finding and contact tracing** | | |
| 12.1 Is there a specific program for tuberculosis case finding at your ART facility? (only one answer)  (If ‘no or unknown’, SKIP to next section done) |      | Yes No  Unknown |
| 12.1.1 If yes, please specify how intensive case finding is conducted (several answers possible) |            | Household contact tracing Community mobilization  Mobile TB diagnostics services offered Door-to-door screening  Other, please specify: ……………………………………………  Unknown |
| 12.2.1. Contact tracing procedures in households when TB is diagnosed at your ART facility (only one answer) |          | Yes No  Sometimes  Other, please specify: ……………………………………………  Unknown |

| **MDR Case Scenario**  You are being asked to participate in the IeDEA MDR-TB Survey. The purpose of this research activity is to study the clinical management of HIV patients co-infected with multi-drug resistant tuberculosis (MDR-TB) or extensively drug-resistant tuberculosis (XDR-TB) from the perspective of health care workers in ART programs participating in the IeDEA global cohort consortium. Completion of the survey will be considered consent to participate.  The Case Scenarios component of the survey is for all sites and is to be completed 3 times independently, by 3 different persons. Respondents must all be health care workers at the ART facility who provide direct care to HIV patients (e.g. medical doctor, clinical officer, nurse, etc.) and may NOT consult with others to answer questions. This component takes about 15 minutes to complete.  Please answer the following questions according to common practice at your clinic. This is not a test and there are no right or wrong answers. | |
| --- | --- |
| **1. Management of TB and MDR-TB at ART facilities** | |
| 1.1. IeDEA Region |  Asia/Pacific   Caribbean, Central and South America   Central Africa   East Africa   Southern Africa   West Africa |
| 1.2. Cohort or Center ID (One answer only) |  …………………………………….. |
| 1.3. Profession of person completing this survey (one answer only)  (If ‘clinical officer or nurse`, SKIP to Q 2.2) |  Medical doctor   Clinical officer   Nurse   Other, please specify: ………………………………………… |
| 1.4. Please indicate your medical specialty, if applicable. (one answer only) |  Infectious diseases   Pulmonology   General Medicine   Pediatrics general   Pediatrics infectious diseases   Pediatrics pulmology   Other, please specify: …………………………………………   N/A |
| **Scenario 1: Adult on a failing first-line TB treatment regimen:**  A 23-year-old man living with HIV who has been on ART for 2 years was diagnosed with TB 3 months ago. He was started on 2HRZE, 4HR and his symptoms improved within a few days. However, his smear microscopy at month 1 was positive and his smear microscopy at month 2 remained positive as well.  What would you do?   1. Request a rapid molecular DST to determine the need to start second-line treatment 2. Refer the patient to the nearest TB facility and follow-up on his progress. 3. Extend the intensive phase of first-line treatment and re-evaluate the patient after one additional month in the intensive phase. 4. Consider the patient to be non-adherent and do not make any treatment changes. Begin the patient on the continuation phase of first-line treatment and counsel him adherence. 5. I don't know   f. Other, please specify: …………………………………………………………………………………………………………………………………………………………………………………… | |
| **Scenario 2: Management of an adult MDR-TB patient:**  A 28-year-old female was recently been diagnosed with HIV. During her evaluation to start ART, she was diagnosed with MDR-TB using Xpert MTB RIF. Her sputum is being cultured on DST solid culture media and the result will be available in two months.  What would you do?   1. Start the patient on a standardized MDR-TB regimen as soon as possible and then start the patient on ART. 2. Wait until the TB facility starts the patient on MDR-TB treatment and then start the patient on ART. 3. Immediately start the patient on ART and refer her to another facility for MDR-TB treatment because 4. MDR-TB drugs are not prescribed at your clinic 5. I don't know 6. Other, please specify: …………………………………………………………………………………………………………………………………………………………………………………… | |
