# Supplementary material for: Integrating services for HIV and multidrug-resistant tuberculosis: A global cross-sectional survey among ART clinics in low- and middle-income countries
Source: PLOS Glob Public Health. 2022 Mar 1;2(3):e0000180. doi: 10.1371/journal.pgph.0000180 (PMC9910322; doi:10.1371/journal.pgph.0000180)
Supplement: S2 Table — Abbreviations: ART, antiretroviral therapy; MDR, multidrug resistance; TB, Tuberculosis. (DOCX) [file pgph.0000180.s002.docx]

**S2 Table:** Infection control measures in place at the 29 ART clinics offering TB services on- and off-site.

|  | **Total**  (n=29, %) | **Asia- Pacific**  (n=6, %) | **South America**  (n=3, %) | **Africa**  (n=20, %) |
| --- | --- | --- | --- | --- |
| **Separate waiting rooms for MDR-TB patients** |  |  |  |  |
| - Yes | 15 (51.7) | 3 (50.0) | 1 (33.3) | 11 (55.5) |
| - No | 14 (48.3) | 3 (50.0) | 2 (66.7) | 9 (45.5) |
| **Separate visiting hours for MDR-TB patients** |  |  |  |  |
| - Yes | 9 (31.0)) | 2 (33.3) | 1 (33.3) | 6 (30.0) |
| - No | 20 (69.0) | 4 (66.7) | 2 (66.7) | 14 (70.0) |
| **Natural air exchange through windows** |  |  |  |  |
| - Yes | 26 (89.7) | 5 (83.3) | 3 (100) | 18 (90.0) |
| - - *Optimized natural ventilation (airflow optimized by size of windows)* | *14 (48.3)* | *4 (66.7)* | *1 (33.3)* | *9 (45.0)* |
| - - *Natural ventilation, but not optimized* | *12 (41.4)* | *1 (16.7)* | *2 (66.7)* | *9 (45.0)* |
| - No natural ventilation | 2 (6.9) | 1 (16.7) | 0 | 1 (5.0) |
| - Unknown | 1 (3.4) | 0 | 0 | 1 (5.0) |
| **Any protection of staff working with TB patients and presumptive TB patients** |  |  |  |  |
| - Regular TB symptom screening (coughing, sweats, fever) | 13 (44.8) | 1 (16.7) | 2 (66.7) | 10 (50.0) |
| - Regular screening by chest X-ray | 13 (44.8) | 4 (66.7) | 2 (66.7) | 7 (35.0) |
| - Regular TB screening by sputum smear regardless of symptoms | 2 (6.9) | 0 | 0 | 2 (10.0) |
| - Regular TB screening by culture regardless of symptoms | 0 | 0 | 0 | 0 |
| - Regular TB screening by molecular tests regardless of symptoms | 1 (3.4) | 0 | 0 | 1 (5.0) |
| - Wearing masks if in close contact to any TB patients | 19 (65.5) | 5 (83.3) | 2 (66.7) | 12 (60.0) |
| - No specific protection measures offered to staff | 4 (13.8) | 1 (16.7) | 1 (33.3) | 2 (10.0) |
| - Unknown | 1 (3.4) | 0 | 1 (33.3) | 0 |

Abbreviations: ART, antiretroviral therapy; MDR, multidrug resistance; TB, Tuberculosis
