# Supplementary material for: Integrating services for HIV and multidrug-resistant tuberculosis: A global cross-sectional survey among ART clinics in low- and middle-income countries
Source: PLOS Glob Public Health. 2022 Mar 1;2(3):e0000180. doi: 10.1371/journal.pgph.0000180 (PMC9910322; doi:10.1371/journal.pgph.0000180)
Supplement: S3 Table — Abbreviations: DST, drug susceptibility testing; DR, drug-resistant; MDR, multidrug resistance; TB, Tuberculosis; XDR, extensive drug resistance. (DOCX) [file pgph.0000180.s003.docx]

**S3 Table:** Availability of TB services by the degree of integration of HIV and MDR-TB services at 29 ART clinics

|  | **Total** |  | **Full integration** |  | **Partial integration** |  | **Off-Site only** |
| --- | --- | --- | --- | --- | --- | --- | --- |
|  | (n=29) |  | (n=14)  n (%) |  | (n=9)  n %) |  | (n=6)  n (%) |
| **Availability of molecular DST for first-line drugs** |  |  |  |  |  |  |  |
| Any on-site | 22 (75.9) |  | 14 (100) |  | 8 (88.9) |  | 0 |
| *Xpert MTB/RIF or Xpert MTB/Ultra* | *21 (72.4)* |  | *14 (100)* |  | *7 (77.8)* |  | *0* |
| *Genotype MTBDRplus* | *3 (10.3)* |  | *2 (14.3)* |  | *1 (11.1)* |  | *0* |
| Any off-site | 12 (42.4) |  | 3 (21.4) |  | 3 (33.3) |  | 6 (100) |
| *Xpert MTB/RIF or Xpert MTB/Ultra* | *7 (24.1)* |  | *0* |  | *1 (11.1)* |  | *6 (100)* |
| *Genotype MTBDRplus* | *7 (24.1)* |  | *3 (21.4)* |  | *3 (33.3)* |  | *1 (16.7)* |
|  |  |  |  |  |  |  |  |
| **Availability of molecular DST for second-line drugs** |  |  |  |  |  |  |  |
| Any on-site | 4 (13.8) |  | 3 (21.4) |  | 1 (11.1) |  | 0 |
| *Xpert MTB/XDR* | *2 (6.9)* |  | *2 (14.3)* |  | *0* |  | *0* |
| *Genotype MTBDRsl* | *3 (10.3)* |  | *2 (14.3)* |  | *1 (11.1)* |  | *0* |
| Any off-site | 11 (37.9) |  | 6 (42.9) |  | 2 (22.2) |  | 3 (50.0) |
| *Xpert MTB/XDR* | *5 (17.2)* |  | *3 (21.4)* |  | *1 (11.1)* |  | *1 (16.7)* |
| *Genotype MTBDRsl* | *6 (20.7)* |  | *3 (21.4)* |  | *1 (11.1)* |  | *2 (33.3)* |
| **Availability of phenotypic DST** |  |  |  |  |  |  |  |
| On-site | 10 (34.5) |  | 7 (50.0) |  | 3 (33.3) |  | 0 |
| *First-line drugs* | *10 (34.5)* |  | *7 (50.0)* |  | *3 (33.3)* |  | *0* |
| *Second-line drugs* | *8 (27.6)* |  | *6 (42.9)* |  | *2 (22.2)* |  | *0* |
| Off-site | 19 (65.5) |  | 7 (50.0) |  | 6 (66.7) |  | 6 (100) |
| *First-line drugs* | *19 (65.5)* |  | *7 (50.0)* |  | *6 (66.7)* |  | *6 (100)* |
| *Second-line drugs* | *8 (27.6)* |  | *3 (21.4)* |  | *3 (33.3)* |  | *2 (33.3)* |
| **DR-TB treatment** |  |  |  |  |  |  |  |
| **MDR-TB treatment** |  |  |  |  |  |  |  |
| On-site | 15 (51.7) |  | 14 (100) |  | 1 (11.1) |  | 0 |
| Off-site | 14 (48.3) |  | 0 |  | 8 (88.9) |  | 6 (100) |
| **MDR-TB regimens** |  |  |  |  |  |  |  |
| Individualised according to the resistance profile | 13 (44.8) |  | 10 (71.4) |  | 2 (22.2) |  | 1 (16.7) |
| Standardised | 11 (37.9) |  | 2 (14.3) |  | 4 (44.4) |  | 5 (83.3) |
| Both (individualised and standardised) | 5 (17.2) |  | 2 (14.3) |  | 3 (33.3) |  | 0 |
| **XDR-TB treatment** |  |  |  |  |  |  |  |
| On-site | 7 (24.1) |  | 6 (42.9) |  | 1 (11.1) |  | 0 |
| Off-site | 22 (75.9) |  | 8 (57.1) |  | 8 (88.9) |  | 6 (100) |

Abbreviations: DST, drug susceptibility testing; DR, drug-resistant; MDR, multidrug resistance; TB, Tuberculosis; XDR, extensive drug resistance
