# Supplementary material for: Integrating services for HIV and multidrug-resistant tuberculosis: A global cross-sectional survey among ART clinics in low- and middle-income countries
Source: PLOS Glob Public Health. 2022 Mar 1;2(3):e0000180. doi: 10.1371/journal.pgph.0000180 (PMC9910322; doi:10.1371/journal.pgph.0000180)
Supplement: S4 Table — Abbreviations: DST, drug susceptibility testing; LAM, urine lipoarabinomannan. * Unknown availability. (DOCX) [file pgph.0000180.s004.docx]

**Supporting Table 4:** On- and off-site availability of diagnostic tests for initial TB diagnosis and diagnosis of drug-resistant TB.

|  |  | **Total** | **On-site**  **n (%)** | **Off-site**  **n (%)** | **Not available**  **n (%)** |
| --- | --- | --- | --- | --- | --- |
| **Initial TB Diagnosis** |  |  |  |  |  |
| *Chest X-ray* |  | 29 | 18 (62.1) | 10 (34.5) | 1 (3.4) |
| *Smear microscopy* |  | 29 | 22 (75.9) | 7 (24.1) | - |
| *Urine LAM* |  | 29 | 7 (24.1) | 2 (6.9) | 20 (69.0) |
| **Molecular DST** |  |  |  |  |  |
| *Any Xpert* |  | 29 | 22 (75.9) | 7 (24.1) | - |
| - *Xpert MTB/RIF* |  | 29 | 21 (72.4) | 7 (24.1) | 1 (3.4) |
| - *Xpert MTB/RIF Ultra* |  | 29 | 7 (24.1) | 3 (10.3) | 19 (65.5) |
| - *Xpert MTB/XDR* |  | 29 | 2 (6.9) | 5 (17.2) | 22 (75.9) |
| *Any line probe assay* |  | 29 | 3 (10.3) | 9 (31.0) | 17 (58.6) |
| - *Genotype MTBDRplus* |  | 29 | 3 (10.3) | 7 (24.1) | 19 (65.5) |
| - *Genotype MTBDRsl* |  | 29 | 3 (10.3) | 6 (20.7) | 20 (69.0) |
| **Phenotypic DST** |  |  |  |  |  |
| *Mycobacterial culture* |  | 29 | 10 (34.5) | 19 (65.5) | - |
| *First-line drugs* |  | 29 | 10 (34.5) | 19 (65.5) | - |
| *Second-line drugs* |  | 29 | 8 (27.6) | 8 (27.6) | 13* (44.8) |

Abbreviations: DST, drug susceptibility testing; LAM, urine lipoarabinomannan

* Unknown availability
