# Supplementary material for: Integrating services for HIV and multidrug-resistant tuberculosis: A global cross-sectional survey among ART clinics in low- and middle-income countries
Source: PLOS Glob Public Health. 2022 Mar 1;2(3):e0000180. doi: 10.1371/journal.pgph.0000180 (PMC9910322; doi:10.1371/journal.pgph.0000180)
Supplement: S5 Table — Abbreviations: ART, antiretroviral therapy; DST, drug susceptibility testing; MDR, multidrug resistance; TB, Tuberculosis; H, isoniazid; R, rifampicin, Z, pyrazinamide; E, ethambutol. (DOCX) [file pgph.0000180.s005.docx]

**S5 Table:** Hypothetical Clinical scenarios assessing clinical practice related to the testing and treatment of MDR-TB at ART-clinics (n=72).

|  | **Total** |  | **Full integration** |  | **Partial integration** |  | **Off-Site only** |
| --- | --- | --- | --- | --- | --- | --- | --- |
|  | (n=72) |  | (n=35)  n (%) |  | (n=24)  n %) |  | (n=13)  n (%) |
| **Scenario 1:**  **A 23-year-old man living with HIV who has been on ART for 2 years was diagnosed with TB 3 months ago. He was started on 2HRZE, 4HR and his symptoms improved within a few days. However, his smear microscopy at month 1 was positive and his smear microscopy at month 2 remained positive as well. What would you do?** |  |  |  |  |  |  |  |
| Request a rapid molecular DST to determine the need to start second-line treatment | 57 (79.2) |  | 31 (88.6) |  | 17 (70.8) |  | 9 (69.2) |
| Extend the intensive phase of first-line treatment and re-evaluate after one month | 6 (8.3) |  | 2 (5.8) |  | 4 (16.7) |  | 0 |
| Consider the patient to be non-adherent and do not make any treatment changes. Begin the patient on the continuation phase of first-line treatment and counsel him adherence. | 4 (5.6) |  | 2 (5.8) |  | 0 |  | 2 (15.4) |
| Refer the patient to a TB clinic | 5 (6.9) |  | 0 |  | 3 (12.5) |  | 2 (15.4) |
| **Scenario 2:**  **A 28-year-old female was recently been diagnosed with HIV. During her evaluation to start ART, she was diagnosed with MDR-TB using Xpert MTB RIF. Her sputum is being cultured on DST solid culture media and the result will be available in two months. What would you do?** |  |  |  |  |  |  |  |
| Start the patient on a standardized MDR-TB regimen as soon as possible and then start the patient on ART. | 58 (80.6) |  | 35 (100) |  | 17 (70.8) |  | 6 (46.2) |
| Wait until the TB facility starts the patient on MDR-TB treatment and then start the patient on ART. | 12 (16.7) |  | 0 |  | 6 (25.0) |  | 6 (46.2) |
| Immediately start the patient on ART and refer her to another facility for MDR-TB treatment because  MDR-TB drugs are not prescribed at your clinic | 2 (2.8) |  | 0 |  | 1 (4.2) |  | 1 (7.7) |

Abbreviations: ART, antiretroviral therapy; DST, drug susceptibility testing; MDR, multidrug resistance; TB, Tuberculosis; H, isoniazid; R, rifiampicin, Z, pyrazinamide; E, ethambutol
