# Supplementary material for: Integrating services for HIV and multidrug-resistant tuberculosis: A global cross-sectional survey among ART clinics in low- and middle-income countries
Source: PLOS Glob Public Health. 2022 Mar 1;2(3):e0000180. doi: 10.1371/journal.pgph.0000180 (PMC9910322; doi:10.1371/journal.pgph.0000180)
Supplement: S7 Table — (DOCX) [file pgph.0000180.s007.docx]

# Regional funding acknowledgements

**IeDEA Asia-Pacific**

## Site investigators and study team:

PS Ly, V Khol, National Center for HIV/AIDS, Dermatology & STDs, Phnom Penh, Cambodia; FJ Zhang, HX Zhao, N Han, Beijing Ditan Hospital, Capital Medical University, Beijing, China; MP Lee, PCK Li, W Lam, HY Wong, Queen Elizabeth Hospital, Hong Kong SAR, China; N Kumarasamy, C Ezhilarasi, Chennai Antiviral Research and Treatment Clinical Research Site (CART CRS), VHS-Infectious Diseases Medical Centre, VHS, Chennai, India; S Pujari, K Joshi, S Gaikwad, A Chitalikar, Institute of Infectious Diseases, Pune, India; TP Merati, DN Wirawan, F Yuliana, Faculty of Medicine Udayana University & Sanglah Hospital, Bali, Indonesia; E Yunihastuti, D Imran, A Widhani, Faculty of Medicine Universitas Indonesia - Dr. Cipto Mangunkusumo General Hospital, Jakarta, Indonesia; J Tanuma, S Oka, T Nishijima, National Center for Global Health and Medicine, Tokyo, Japan; JY Choi, Na S, JM Kim, Division of Infectious Diseases, Department of Internal Medicine, Yonsei University College of Medicine, Seoul, South Korea; YM Gani, NB Rudi, Hospital Sungai Buloh, Sungai Buloh, Malaysia; I Azwa, A Kamarulzaman, SF Syed Omar, S Ponnampalavanar, University Malaya Medical Centre, Kuala Lumpur, Malaysia; R Ditangco, MK Pasayan, ML Mationg, Research Institute for Tropical Medicine, Muntinlupa City, Philippines; YJ Chan, WW Ku, E Ke, PC Wu, Taipei Veterans General Hospital, Taipei, Taiwan; OT Ng, PL Lim, LS Lee, JK Yap, Tan Tock Seng Hospital, Singapore; A Avihingsanon, S Gatechompol, P Phanuphak, C Phadungphon, HIV-NAT/Thai Red Cross AIDS Research Centre, Bangkok, Thailand; S Kiertiburanakul, A Phuphuakrat, L Chumla, N Sanmeema, Faculty of Medicine Ramathibodi Hospital, Mahidol University, Bangkok, Thailand; R Chaiwarith, T Sirisanthana, J Praparattanapan, K Nuket, Research Institute for Health Sciences, Chiang Mai, Thailand; S Khuwuwan, P Kantipong, P Kambua, Chiangrai Prachanukroh Hospital, Chiang Rai, Thailand; KV Nguyen, HV Bui, DTH Nguyen, DT Nguyen, National Hospital for Tropical Diseases, Hanoi, Vietnam; CD Do, AV Ngo, LT Nguyen, Bach Mai Hospital, Hanoi, Vietnam; AH Sohn, JL Ross, B Petersen, TREAT Asia, amfAR - The Foundation for AIDS Research, Bangkok, Thailand; MG Law, A Jiamsakul, R Bijker, D Rupasinghe, The Kirby Institute, UNSW Sydney, NSW, Australia.

# CCASAnet

## IeDEA Caribbean, Central, and South America (CCASAnet):

Fundación Huésped, Argentina: Pedro Cahn, Carina Cesar, Valeria Fink, Omar Sued, Emanuel Dell’Isola, Hector Perez, Jose Valiente, Cleyton Yamamoto; Instituto Nacional de Infectologia-Fiocruz, Brazil: Beatriz Grinsztejn, Valdilea Veloso, Paula Luz, Raquel de Boni, Sandra Cardoso Wagner, Ruth Friedman, Ronaldo Moreira. Universidade Federal de Minas Gerais, Brazil: Jorge Pinto, Flavia Ferreira, Marcelle Maia; Universidade Federal de São Paulo, Brazil: Regina Célia de Menezes Succi, Daisy Maria Machado, Aida de Fátima Barbosa Gouvêa; Fundación Arriarán, Chile: Marcelo Wolff, Claudia Cortes, Maria Fernanda Rodriguez, Gladys Allendes; Les Centres GHESKIO, Haiti: Jean William Pape, Vanessa Rouzier, Adias Marcelin, Christian Perodin; Hospital Escuela Universitario, Honduras: Marco Tulio Luque; Instituto Hondureño de Seguridad Social, Honduras: Denis Padgett; Instituto Nacional de Ciencias Médicas y Nutrición Salvador Zubirán, Mexico: Juan Sierra Madero, Brenda Crabtree Ramirez, Paco Belaunzaran, Yanink Caro Vega; Instituto de Medicina Tropical Alexander von Humboldt, Peru: Eduardo Gotuzzo, Fernando Mejia, Gabriela Carriquiry

**Vanderbilt University Medical Center, USA:**

Catherine C McGowan, Bryan E Shepherd, Timothy Sterling, Karu Jayathilake, Anna K Person, Peter F Rebeiro, Jessica Castilho, Stephany N Duda, Fernanda Maruri, Hilary Vansell, Cathy Jenkins, Ahra Kim, Sarah Lotspeich.

# Central Africa (CA-IeDEA)

**Site investigators and cohorts:**

Nimbona Pélagie, Association Nationale de Soutien aux Séropositifs et Malade du Sida (ANSS), Burundi; Patrick Gateretse, Jeanine Munezero, Valentin Nitereka, Théodore Niyongabo, Christelle Twizere, Centre National de Référence en Matière de VIH/SIDA, Burundi; Hélène Bukuru, Thierry Nahimana, Centre de Prise en Charge Ambulatoire et Multidisciplinaire des PVVIH/SIDA du Centre Hospitalo-Universitaire de Kamenge (CPAMP- CHUK), Burundi; Elysée Baransaka, Patrice Barasukana, Eugene Kabanda, Martin Manirakiza, François Ndikumwenayo, CHUK/Burundi National University, Burundi; Jérémie Biziragusenyuka, Ange Marie Michelline Munezero, Centre de Prise en Charge Ambulatoire et Multidisciplinaire des PVVIH/SIDA de l’Hôpital Prince Régent Charles (CPAMP-HPRC), Burundi; Denis Nsame Nforniwe, Bamenda Hospital, Cameroon; Rogers Ajeh, Marc Lionel Ngamani, Clinical Research Education and Consultancy (CRENC), Cameroon; Anastase Dzudie, CRENC and Douala General Hospital, Cameroon; Akindeh Mbuh, CRENC and University of Yaoundé, Cameroon; Djenabou Amadou, Eric Walter Pefura Yone, Jamot Hospital, Cameroon; Ernestine Kendowo, Limbe Regional Hospital, Cameroon; Catherine Akele, Akili Clever, Faustin Kitetele, Patricia Lelo, Martine Tabala, Kalembelembe Pediatric Hospital, Democratic Republic of Congo; Cherubin Ekembe, Didine Kaba, Kinshasa School of Public Health, Democratic Republic of Congo; Merlin Diafouka, Martin Herbas Ekat, Dominique Mahambou Nsonde, CTA Brazzaville, Republic of Congo; Adolphe Mafoua, Massamba Ndala Christ, CTA Pointe-Noire, Republic of Congo; Jules Igirimbabazi, Nicole Ayinkamiye, Bethsaida Health Center, Rwanda; Providance Uwineza, Emmanuel Ndamijimana, Busanza Health Center, Rwanda; Emmanuel Habarurema, Marie Luise Nyiraneza, Gahanga Health Center, Rwanda; Marie Louise Nyiransabimana, Liliane Tuyisenge, Gikondo Health Center, Rwanda; Christian Shyaka, Catherine Kankindi, Kabuga Health Center, Rwanda; Bonheur Uwakijijwe, Marie Grace Ingabire, Kicukiro Health Center, Rwanda; Jules Ndumuhire, Marie Goretti Nyirabahutu, Masaka Health Center, Rwanda; Fred Muyango, Jean Christophe Bihibindi, Nyagasambu Health Center, Rwanda; Oliver Uwamahoro, Yvette Ndoli, Nyarugunga Health Center, Rwanda; Sabin Nsanzimana, Placidie Mugwaneza, Eric Remera, Esperance Umumararungu, Gallican Nshogoza Rwibasira, Dominique Savio Habimana, Rwanda Biomedical Center, Rwanda; Josephine Gasana, Faustin Kanyabwisha, Gallican Kubwimana, Benjamin Muhoza, Athanase Munyaneza, Gad Murenzi, Francoise Musabyimana, Francine Umwiza, Charles Ingabire, Patrick Tuyisenge, Alex M. Butera, Jules Kabahizi, Ephrem Rurangwa, Rwanda Military Hospital, Rwanda; Rosine Feza, Eugenie Mukashyaka, Shyorongi Health Center, Rwanda; Chantal Benekigeri, Jacqueline Musaninyange, WE-ACTx Health Center, Rwanda.

## Coordinating and Data Centers:

Adebola Adedimeji, Kathryn Anastos, Madeline Dilorenzo, Lynn Murchison, Jonathan Ross, Marcel Yotebieng, Albert Einstein College of Medicine, USA; Diane Addison, Ellen Brazier, Heidi Jones, Elizabeth Kelvin, Sarah Kulkarni, Denis Nash, Matthew Romo, Olga Tymejczyk, Institute for Implementation Science in Population Health, Graduate School of Public Health and Health Policy, City University of New York (CUNY), USA; Batya Elul, Columbia University, USA; Xiatao Cai, Allan Dong, Don Hoover, Hae-Young Kim, Chunshan Li, Qiuhu Shi, Data Solutions, USA; Kathryn Lancaster, The Ohio State University, USA; Mark Kuniholm, University at Albany, State University of New York, USA; Andrew Edmonds, Angela Parcesepe, Jess Edwards, University of North Carolina at Chapel Hill, USA; Olivia Keiser, University of Geneva; Stephany Duda; Vanderbilt University School of Medicine, USA; April Kimmel, Virginia Commonwealth University School of Medicine, USA.

# EAST AFRICA IeDEA

## Site investigators and cohorts (no data managers)

Lameck Diero, Samuel Ayaya (Academic Model Providing Access to Healthcare (AMPATH), Eldoret, Kenya), Elizabeth Bukusi (Family [Family AIDS Care & Education Services](https://faces.ucsf.edu/) (FACES), Kisumu, Kenya), Charles Kasozi (Masaka Regional Referral Hospital, Masaka, Uganda), Mwebesa Bosco Bwana (Mbarara University of Science and Technology (MUST), Winnie Muyindike, Helen Byakwaga (Mbarara, Uganda), Barbara Castelnuovo, Aggrey Semeere, (Infectious Diseases Institute (IDI), Kampala, Uganda), Fred Nalugoda (Rakai Health Sciences Program (RHSP), Kalisizo, Uganda), Paul Kazyoba, Mary Mayige, (NIMR), Dar es Salaam, Tanzania), Rita Elias Lyamuya, Regional Hospital, Morogoro, Tanzania Kapella Ngonyani, Tumbi Regional Hospital, Pwani, Tanzania; and Mark Urassa , Charles Nyaga (National Institute for Medical Research (NIMR), Mwanza, Tanzania), Batya Elul (Columbia University, New York, USA), Rachel Vreeman (Mt. Sinai, New York, USA) Jennifer Syvertsen (University of California Riverside, California, USA) Rami Kantor (Brown University, Providence, USA), Jeff Martin (University of California, San Francisco, USA), Craig Cohen (University of California, San Francisco, USA), East Africa IeDEA Regional Data Center, Indiana University: Kara Wools-Kaloustian and Constantin Yiannoutsos.

## Site investigators and cohorts (with data managers):

Diero L, Ayaya S, Sang E, MOI University, AMPATH Plus, Eldoret, Kenya; Bukusi E, Edwin Mulwa, George Nyanaro, KEMRI (Kenya Medical Research Institute), Kisumu, Kenya; Charles Kasozi , Mathew Ssemakadde, Masaka Regional Referral Hospital, Masaka, Uganda; Mwebesa Bosco Bwana, Winnie Muyindike, Helen Byakwaga Michael Kanyesigye, Mbarara University of Science and Technology (MUST), Mbarara, Uganda; Barbara Castelnuovo, Aggrey Semeere,; John Michael Matovu, Infectious Diseases Institute (IDI), Mulago, Uganda; Fred Nalugoda, Francis X. Wasswa, Rakai Health Sciences Program, Kalisizo, Uganda; Paul Kazyoba, Mary Mayige, (NIMR), Dar es Salaam, Tanzania; Rita Elias Lyamuya, Francis Mayanga, Morogoro Regional Hospital, Morogoro, Tanzania; Kapella Ngonyani, Jerome Lwali, Tumbi Regional Hospital, Pwani, Tanzania; Mark Urassa, Charles Nyaga, Richard Machemba, National Institute for Medical Research (NIMR), Kisesa HDSS, Mwanza, Tanzania; Kara Wools-Kaloustian, Constantin Yiannoutsos, Beverly Musick, Indiana University School of Medicine, Indiana University, Indianapolis, IN, USA; Batya Elul, Columbia University, New York City, NY, USA; Rachel Vreeman (Mt. Sinai, New York, USA) Jennifer Syvertsen, (University of California Riverside, California, USA; Rami Kantor, Brown University/Miriam Hospital, Providence, RI, USA; Jeffrey Martin, Megan Wenger, Craig Cohen, Jayne Kulzer, University of California, San Francisco, CA, USA;

# IeDEA Southern Africa

## Site investigators and cohorts:

Gary Maartens, Aid for AIDS, South Africa; Carolyn Bolton, Centre for Infectious Disease Research in Zambia (CIDRZ), Zambia; Robin Wood, Gugulethu (Desmond Tutu HIV Centre), South Africa; Nosisa Sipambo, Harriet Shezi Children’s Clinic, South Africa; Frank Tanser, Hlabisa (Africa Health Research Institute), South Africa; Andrew Boulle, Khayelitsha ART Programme, South Africa; Geoffrey Fatti, Kheth’Impilo AIDS Free Living, South Africa; Safari Mbewe, Lighthouse Trust, Malawi; Elvira Singh, National Cancer Registry (National Health Laboratory Service), South Africa; Cleophas Chimbetete, Newlands Clinic (Ruedi Luethy Foundation Zimbabwe), Zimbabwe; Karl Technau, Rahima Moosa Mother and Child Hospital, South Africa; Brian Eley, Red Cross War Memorial Children’s Hospital, South Africa; Josephine Muhairwe, SolidarMed Lesotho; Idivino Rafael, SolidarMed Mozambique; Cordelia Kunzekwenyika, SolidarMed Zimbabwe, Matthew P Fox, Themba Lethu Clinic, South Africa; Hans Prozesky, Tygerberg Hospital, South Africa; Andrew Boule, Western Cape Provincial Health Data Centre.

## Data centers:

Nina Anderegg, Marie Ballif, Cam Ha Dao Ostinelli, Matthias Egger, Lukas Fenner, Andreas Haas, Stefanie Hossmann, Radoslaw Panczak, Eliane Rohner, Julien Riou, Veronika W Skrivankova, Lilian Smith, Katayoun Taghavi, Per von Groote, Gilles Wandeler, Anja Wettstein, Elizabeth Zaniewski, Kathrin Zürcher, Institute of Social and Preventive Medicine, University of Bern, Switzerland; Kim Anderson, Andrew Boulle, Chido Chinogurei, Morna Cornell, Mary-Ann Davies, Victoria Iyun, Leigh Johnson, Reshma Kassanjee, Kathleen Kehoe, Mmamapudi Kubjane, Nicola Maxwell, Carl Morrow, Patience Nyakato, Gem Patten, Mpho Tlali, Priscilla Tsondai, Renee de Waal, School of Public Health and Family Medicine, University of Cape Town, South Africa.

# West Africa

## Site investigators and cohorts:

Adult cohorts: Marcel Djimon Zannou, CNHU, Cotonou, Benin; Armel Poda, CHU Souro Sanou, Bobo Dioulasso, Burkina Faso; Fred Stephen Sarfo & Komfo Anokeye Teaching Hospital, Kumasi, Ghana; Eugene Messou, ACONDA CePReF, Abidjan, Cote d’Ivoire; Henri Chenal, CIRBA, Abidjan, Cote d’Ivoire; Kla Albert Minga, CNTS, Abidjan, Cote d’Ivoire; Emmanuel Bissagnene, & Aristophane Tanon, CHU Treichville, Cote d’Ivoire; Moussa Seydi, CHU de Fann, Dakar, Senegal; Akessiwe Akouda Patassi, CHU Sylvanus Olympio, Lomé,

## Coordinating & data centers:

François Dabis, Elise Arrive, Eric Balestre, Renaud Becquet, Charlotte Bernard, Shino Chassagne Arikawa, Alexandra Doring, Antoine Jaquet, Karen Malateste, Elodie Rabourdin, Thierry Tiendrebeogo, ADERA, Isped & INSERM U1219, Bordeaux, France.

Sophie Desmonde, Julie Jesson, Valeriane Leroy, Inserm 1027, Toulouse, France

Didier Koumavi Ekouevi, Jean-Claude Azani, Patrick Coffie, Abdoulaye Cissé, Guy Gnepa, Apollinaire Horo, Christian Kouadio, Boris Tchounga, PACCI, CHU Treichville, Abidjan, Côte d’Ivoire

## Coordinating & data centers:

François Dabis, Elise Arrive, Eric Balestre, Renaud Becquet, Charlotte Bernard, Shino Chassagne Arikawa, Alexandra Doring, Antoine Jaquet, Karen Malateste, Elodie Rabourdin, Thierry Tiendrebeogo, ADERA, Isped & INSERM U1219, Bordeaux, France.

Sophie Desmonde, Julie Jesson, Valeriane Leroy, Inserm 1027, Toulouse, France

Didier Koumavi Ekouevi, Jean-Claude Azani, Patrick Coffie, Abdoulaye Cissé, Guy Gnepa, Apollinaire Horo, Christian Kouadio, Boris Tchounga, PACCI, CHU Treichville, Abidjan, Côte d’Ivoire
